# Organophosphorus Flame Retardant, Phthalate, and Alternative Plasticizer Contamination in Novel Plant-Based Food: A Food Safety Investigation

**DOI:** 10.1021/acs.est.4c11805

**Published:** 2025-03-21

**Authors:** Alicia Macan Schönleben, Fatima den Ouden, Shanshan Yin, Erik Fransen, Stijn Bosschaerts, Mirjana Andjelkovic, Nayyer Rehman, Alexander L. N. van Nuijs, Adrian Covaci, Giulia Poma

**Affiliations:** † Toxicological Centre, University of Antwerp, Universiteitsplein 1, 2610 Wilrijk, Belgium; ‡ Key Laboratory of Pollution Exposure and Health Intervention of Zhejiang Province, Interdisciplinary Research Academy (IRA), Zhejiang Shuren University, Hangzhou 310015, China; § Centre of Medical Genetics, University of Antwerp and Antwerp University Hospital, 2650 Edegem, Belgium; # Centre for Oncological Research, University of Antwerp and Antwerp University Hospital, 2610 Wilrijk, Belgium; ∥ Sciensano, Service Risk and Health Impact Assessment, Juliette Wytsmanstraat 14, 1050 Brussels, Belgium; ⊥ WRG Europe Ltd., 26-28 Southernhay East, EX1 1NS, Exeter, U.K.

**Keywords:** Emerging contaminants, Vegan diet, Alternative Proteins, Exposome, Human exposure, Risk assessment

## Abstract

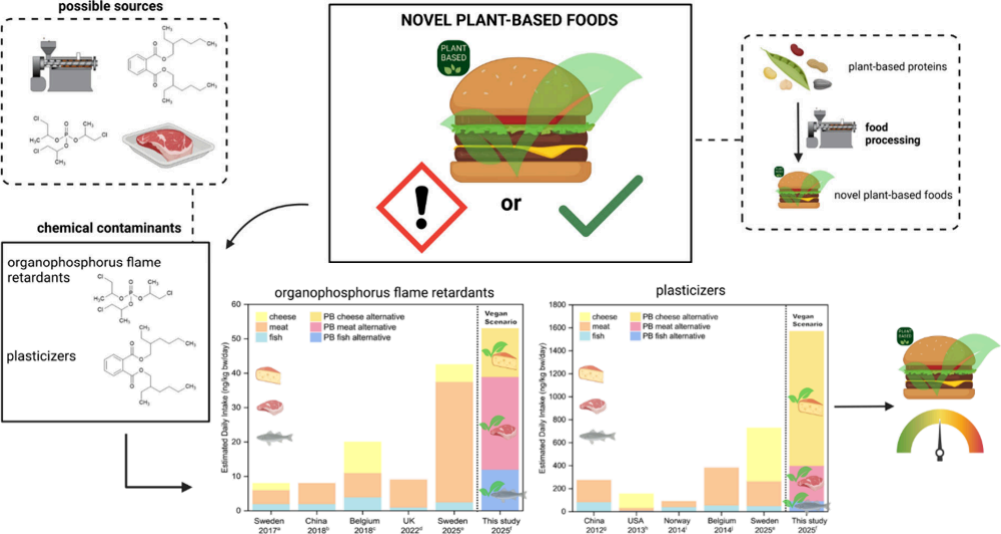

With plant-based (PB) diets gaining popularity, ultraprocessed novel plant-based foods (NPBFs) are an increasingly available alternative to animal-based foods (ABFs). The degree of industrial food processing has been associated with higher organophosphorus flame retardant (PFR) and plasticizer contamination. Here, the occurrence of these contaminants in NPBFs was investigated by using liquid chromatography-tandem mass spectrometry. Our findings show differences in contamination levels and patterns between PB food categories, with PB cheese-alternatives showing the highest levels of both total PFRs (mean: 123 ng/g ww) and total plasticizers (mean: 1155 ng/g ww). The results further point to food contact material and industrial processing as possible contamination sources. Compared with previous studies of ABFs, NPBFs generally showed higher contamination levels, leading to a higher dietary exposure in a vegan diet scenario. While the adult population is not at immediate risk following NPBF consumption, based on these results, a direct replacement of all ABFs with NPBFs is not recommended. Additionally, it is suggested that different PB food categories be included in future food studies monitoring dietary exposure.

## Abstract 2

Analysis of organophosphorus flame retardants and plasticizers in novel plant-based foods revealed different contamination levels between food categories and showed higher exposure compared to animal-based products.

## 1 Introduction

In recent years, plant-based (PB) diets (such as vegetarianism and veganism) have become increasingly popular in Western countries, mostly driven by environmental ethics or health concerns. Plant-based foods have been shown to produce only 50% of greenhouse-gas (GHG) emissions compared to animal-based foods (ABFs),^1^ and vegan diets could reduce GHG emissions, water pollution, and land use by 75% compared to meat-rich diets.^2^ PB diets are also currently considered healthier and have been associated with a lower risk of developing various diseases,^3−5^ such as cardiovascular diseases,^3,4,6^ diabetes, and obesity.^7^ However, these positive effects are mostly linked to an increased consumption of healthy food, including fresh vegetables and fruits, while not all PB diets necessarily have beneficial health effects.^8^ For example, poorly formulated vegan diets, consisting of low-quality ultraprocessed foods (UPFs) rich in refined carbohydrates, saturated fats, and added sugars, have even been already associated with adverse health effects.^8−10^

Along with the gained popularity of PB diets, the demand for novel plant-based foods (NPBFs) and their market are steeply increasing. NPBFs refers to PB (i.e., vegan and vegetarian) alternatives that intend to replace animal products (such as meat or cheese) without using animal-originated ingredients. This has resulted not only in companies and startups specifically focusing on the development of NPBFs but also in large companies introducing vegan and vegetarian alternatives.^11^ The sales of PB food in the US currently reached 8 billion dollars, while in Europe the PB food market is already worth 5.8 billion Euro, with Germany (1.9 billion Euro in 2022) and the United Kingdom (UK) (964 million in 2022) owning the biggest share.^12^ However, to reach a desired end-product that imitates ABFs, these products often need to undergo substantial industrial processing (Figure S1, Section S1) to achieve a comparable texture, taste, and shape of e.g. meat or cheese, placing most of them in category 4 (UPFs) of the NOVA classification system.^13,14^

Two important chemical classes associated with food processing are organophosphorus flame retardants (PFRs) and plasticizers, including legacy phthalates (LPs) and alternative plasticizers (APs).^15,16^ These chemicals are added to materials to enhance their resistance to fire, in the case of PFRs,^17^ and to increase product durability, flexibility, and elasticity, in the case of plasticizers.^18^ While there is still uncertainty about their toxicity, several PFRs and plasticizers have been shown to cause adverse health effects such as neurotoxicity, carcinogenicity,^19^ reproductive toxicity, and endocrine disruption.^20−22^ Since these chemicals are used as additives and are not chemically bound to the products, they are prone to be released into the environment and food.^17^ Contaminated food, in particular, has been identified as a major route for human exposure to PFRs and plasticizers.^23−25^ For both compound classes, processed food showed to be highly contaminated, suggesting industrial processing and migration from food contact materials (FCMs) as possible sources.^15,16,26^ While most studies currently focus on the environmental impact^1,2,27^ or consumer perception toward NPBFs^28^ and PB diets, information about the chemical contamination and safety of such products is still scarce.

In the present study, the contamination levels and patterns of selected PFRs, LPs, and APs in 52 processed and ultraprocessed NPBFs from three European countries were investigated, and possible sources of contamination, such as FCMs and food processing, were hypothesized. Further, the chemical safety of the analyzed NPBFs was evaluated by performing a dietary exposure risk assessment in the frame of vegan, vegetarian, and flexitarian diet scenarios.

## 2 Methods

### 2.1 Sampling

A total of 52 NPBFs were purchased in March–April 2023 from different large grocery chains in Belgium, Germany, and the UK, including a large selection of commercial brands (Supporting Information, Table S1). To ensure anonymity, stores as well as brands were coded. In brief, the samples were sorted in the following categories according to the label of the purchased product: PB meat-alternatives (including PB burger-, chicken-, minced meat-, sausage-, and cold meat-alternatives and processed soy products such as tofu), PB cheese-alternatives, PB fish-alternatives, and various, which included items which could not be placed in any of the previous categories (i.e., drumsticks, meatballs, salami, and a beet burger). The NPBFs are based on different plant ingredients like soy, legumes, vegetables, grains, mycoprotein, oils, and seeds and nuts. All NPBF samples were cut into small pieces using precleaned utensils, freeze-dried, and individually homogenized using a mortar, to avoid the use of electric blenders which might introduce a certain degree of external contamination. Samples were stored in precleaned PP tubes pending analysis.

Since PFRs, LPs, and APs are used as additives in plastics, and to investigate possible migration from the packaging to the food, the corresponding food contact materials were also individually collected, coded (Table S1), and cut into pieces of about 1–2 cm^2^ using precleaned utensils prior to analysis.

### 2.2 Chemical Analysis

#### 2.2.1 PFRs, LPs, and APs in NPBFs

Detailed information about purchased chemicals and materials is reported in Table S2.

For the extraction of NPBFs, sample preparation was performed according to Poma et al.^29^ Each dry sample (0.10–0.15 g) was weighed in a precleaned glass tube and spiked with 50 μL of internal standard (IS) mixtures for PFRs (2 ng/μL TBOEP-d6, TCEP-d12, TDCIPP-d15, and TPHP-d15) and plasticizers (10 ng/μL DEHP-d4, DNBP-d4, and DBzP-d4). A 5 mL mixture of acetonitrile and toluene (9:1 v/v) was added, and the sample was vortexed for 1 min, sonicated for 5 min, and centrifuged at 3000 rpm for 3 min. The supernatant was transferred to a new glass tube and concentrated to 2 mL under a gentle nitrogen flow. For cleanup, dispersive solid phase extraction (d-SPE) was performed by adding 50 mg of primary secondary amine and 100 mg of C18, followed by 1 min of vortexing and 3 min of centrifugation at 3000 rpm. The supernatant was transferred to a new glass tube, evaporated to dryness, and finally reconstituted in 1 mL of hexane. The solution was then loaded on a Florisil cartridge (preconditioned with 4 mL of acetone, 6 mL of ethyl acetate, and 6 mL of hexane). The fractionation was achieved with 12 mL of hexane:dichloromethane (4:1 v/v) (F1) and 10 mL of ethyl acetate and 8 mL of acetone (F2). F1 was discarded, while F2 was concentrated to near dryness using nitrogen. The samples were reconstituted in 50 μL of methanol and 50 μL of recovery standard (RS, triamyl phosphate, 1 ng/μL in methanol) and filtered through a 0.2 μm centrifugal filter.

A volume of 15 μL of the final extract was aliquoted to an amber injection vial, and 135 μL of ethyl acetate was added for quantitative analysis of bis(2-ethylhexyl) phthalate (DEHP) and bis(2-ethylhexyl) terephthalate (DEHT) by GC/MS, as they have similar MRM transitions for LC-MS/MS and cannot be separated by most LC columns. The remaining aliquots were transferred to an autosampler vial for LC-MS/MS analysis. They were left at −20 °C overnight to check for lipid precipitation and filtered again if necessary. Details of the instrumental analysis for GC/MS and LC-MS/MS are reported in the SI (Section S2).

#### 2.2.2 PFRs, LPs, and APs in FCMs

Samples were prepared according to Poma et al.^29^ with minor modifications, using acetonitrile instead of hexane for extraction. Briefly, each FCM was cut into 3 × 3 mm pieces, and ∼50 mg was weighed and sonicated with 1 mL of acetonitrile for 60 min. The supernatant was transferred to a new glass tube, and the extraction was repeated using 1 mL of fresh solvent. The extracts were combined and evaporated to dryness using nitrogen. The samples were reconstituted in 50 μL of methanol and 50 μL of RS (TAP, 1 ng/μL), filtered through a 0.2 μm centrifugal filter, and analyzed as described above.

### 2.3 Quality Assurance and Quality Control

For the determination of repeatability and recovery of the analytical method, quality control samples (QC) were included in each run (PFRs were spiked at 5 ng, while PHs and APs were spiked at 200 ng). QC accuracies were generally within the range of acceptability (75–125%) (Table S3). The mean IS recoveries in the NPBF samples ranged from 52 to 93% for PFRs and from 81 to 119% for plasticizers. In FCM, they ranged from 80 to 92% for PFRs and from 70 to 82% for plasticizers. To control potential background contamination, two procedural blanks (prebaked Na_2_SO_4_) were run in parallel with each batch (n = 21) of samples, and an additional two were included during the freeze-drying process. To avoid background contamination from dust and reduce analyte levels in the procedural blanks, precautionary cleaning steps were included, such as baking glass equipment at 300 °C for 2.5 h. Even though these precautionary measures were taken, several contaminants could still be detected in the blank samples. To achieve representative limits of quantification (LOQs), average blank concentrations were therefore calculated separately for each batch and then subtracted from the concentrations in the samples. LOQs were calculated by using three times the standard deviation (SD) of the blank measurements of each batch (ng/g ww for NPBFs, ng/g plastic for FCM) (Table S3).

### 2.4 Data Analysis and Statistical Analysis

For data processing, values < LOQ were treated as LOQ × DF where DF is the detection frequency of the compound in the samples above LOQ.^30^ Statistical analysis was performed with IBM SPSS 20 (Chicago, Illinois, USA) and R version 4.3.2. Associations between fat content and contamination levels of individual compounds were tested for significance using regression with fat content as the dependent variable and the logarithm of the contaminant’s concentration as the dependent variable. Correlations between NPBF and corresponding FCM were expressed by Spearman correlation (non-normality) and split either by food category or by base ingredient. Only PB food categories with *n* > 3 samples were considered for statistical analysis.

### 2.5 Dietary Exposure and Risk Assessment

**Figure 1:**
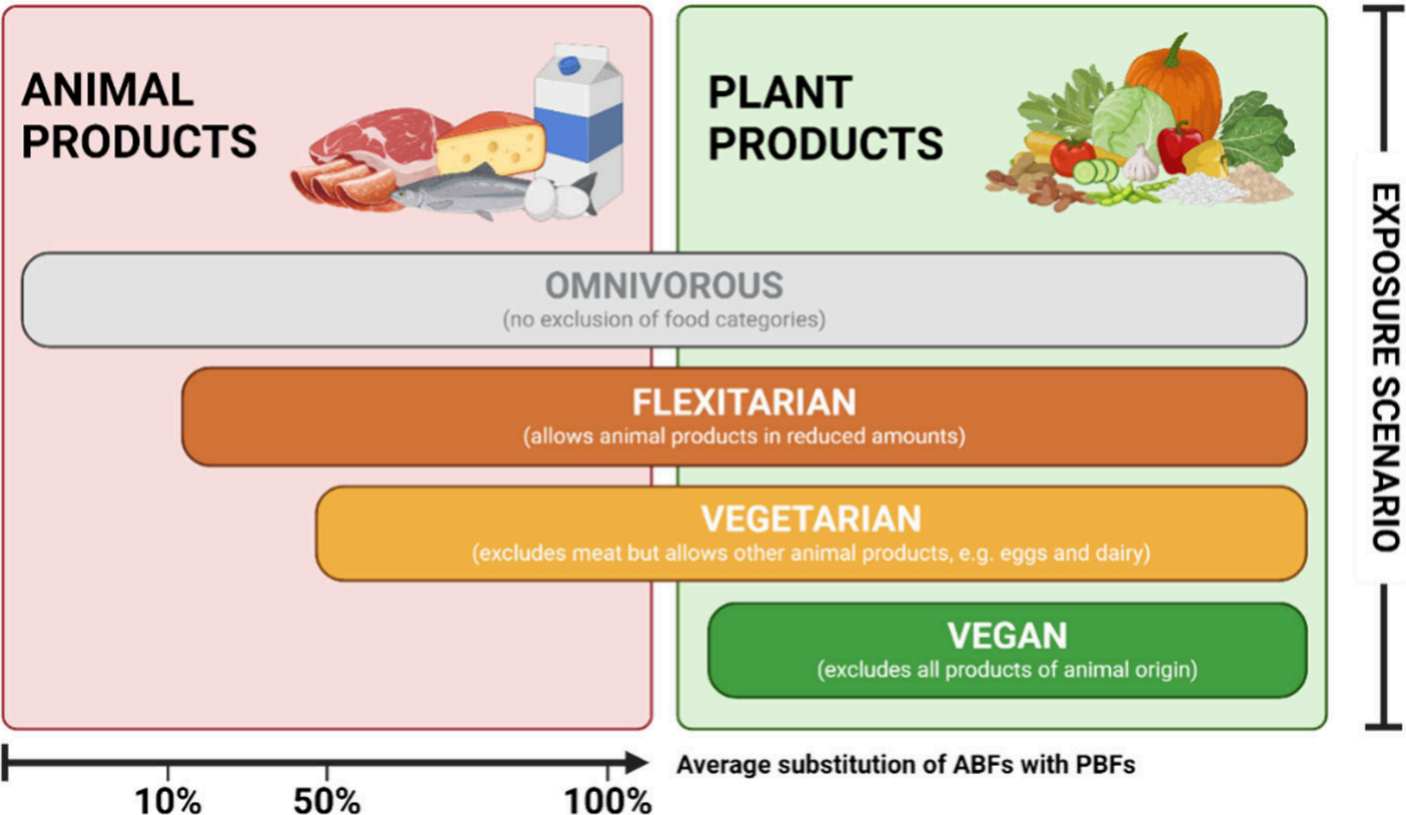
Overview of different dietary patterns and assumptive exposure scenarios. Definitions for dietary patterns were proposed by Hargreaves et al., 2023.^31^ Three different assumptive exposure scenarios were calculated for a vegan diet, vegetarian diet, and flexitarian diet. Due to the lack of clear definitions,^31^ it was assumed a vegan would consume 100% NPBFs (assigning average meat, fish, and cheese consumption to PB meat-, fish-, and cheese-alternatives) to substitute an animal-protein based diet. A vegetarian would consume 50% NPBFs (assigning average meat consumption to PB meat-alternatives) which are supplemented with a certain number of ABFs (like eggs and cheese), while a flexitarian would consume 10% NPBFs (assigning average meat consumption to PB meat-alternatives) in addition to ABFs. Created in BioRender. Poma, G. (2024) BioRender.com/x85v119.

The estimated daily intake (EDI) for PFRs and plasticizers was calculated by multiplying the median concentration (ng/g ww) of each compound by the average daily consumption rate (g per day) of the adult population (18–64 years). Since specific data regarding NPBF consumption are not available yet, EDIs were calculated using three different assumptive scenarios: vegan diet, vegetarian diet, and flexitarian diet (Figure 1). Due to the lack of clear definitions,^31^ it was assumed that in the vegan scenario 100% of average meat, fish, and cheese consumption was substituted with PB meat-, fish-, and cheese-alternatives to represent the maximum intake of NPBFs and therefore approximate a worst-case scenario; in the vegetarian scenario 50% of average meat consumption was substituted with PB meat-alternatives (assuming that vegetarians replace meat consumption with NPBFs 3–4 times per week); and in the flexitarian scenario 10% of average meat consumption was substituted with PB meat-alternatives (assuming that flexitarians replace meat consumption with NPBFs 1 time per week). For the vegetarian and flexitarian scenario, only meat products were replaced by PB meat-alternatives, as it was assumed that vegetarians would still supplement their diet with a certain type of ABFs (like eggs and cheese) and flexitarians would still consume ABFs but in lower amounts and supplemented with NPBFs. Therefore, the vegan scenario represents the worst-case scenario in terms of human exposure to NPBFs.

The consumption data for animal products, including meat, fish, and cheese, were obtained for the three countries where the samples were purchased from, namely Belgium, Germany, and the UK. For Belgium and the UK, the consumption data was derived from the European Food Safety Authority Consumption database,^32^ while for Germany, the data was derived from the German Federal Ministry of Food and Agriculture.^33^ However, the UK data were rather outdated (2008), and it was assumed that the consumption rate of the selected food has somewhat changed since then; therefore, these data were not considered for further calculations. Additionally, since the consumption patterns of Belgium (from 2014) and Germany (from 2023) were comparable (Table S4), the German consumption data was used as a proxy to further calculate EDIs in the frame of this study and compare them with previous studies. The EDI values (ng/day) were then divided by 70 kg body weight (bw)^34^ to obtain EDI values in ng/kg bw/day.

The risk characterization ratio (RCR) for individual compounds was calculated according to ECHA^35^
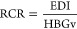
where the EDI (ng/kg bw/day) was calculated as described above, and HBGV is the most conservative available health-based guidance value (ng/kg bw/day).^36,37^ If no HBGV was available, the RCR was calculated as
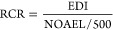
where NOAEL is the most conservative available no-observed adverse effect level or benchmark dose level (BMDL),^36,37^ and the factor 500 was selected based on the default value 100 multiplied by a factor 5, to account for the uncertainties in the data set as well as the lack of toxicological values.^34^ Calculated RCR values ≤ 1 indicate that potential health risks for the adult population resulting from the exposure to that compound can be considered unlikely.^35^

## 3 Results and Discussion

### 3.1 PFRs, LPs, and APs in Novel Plant-Based Foods

**Figure 2:**
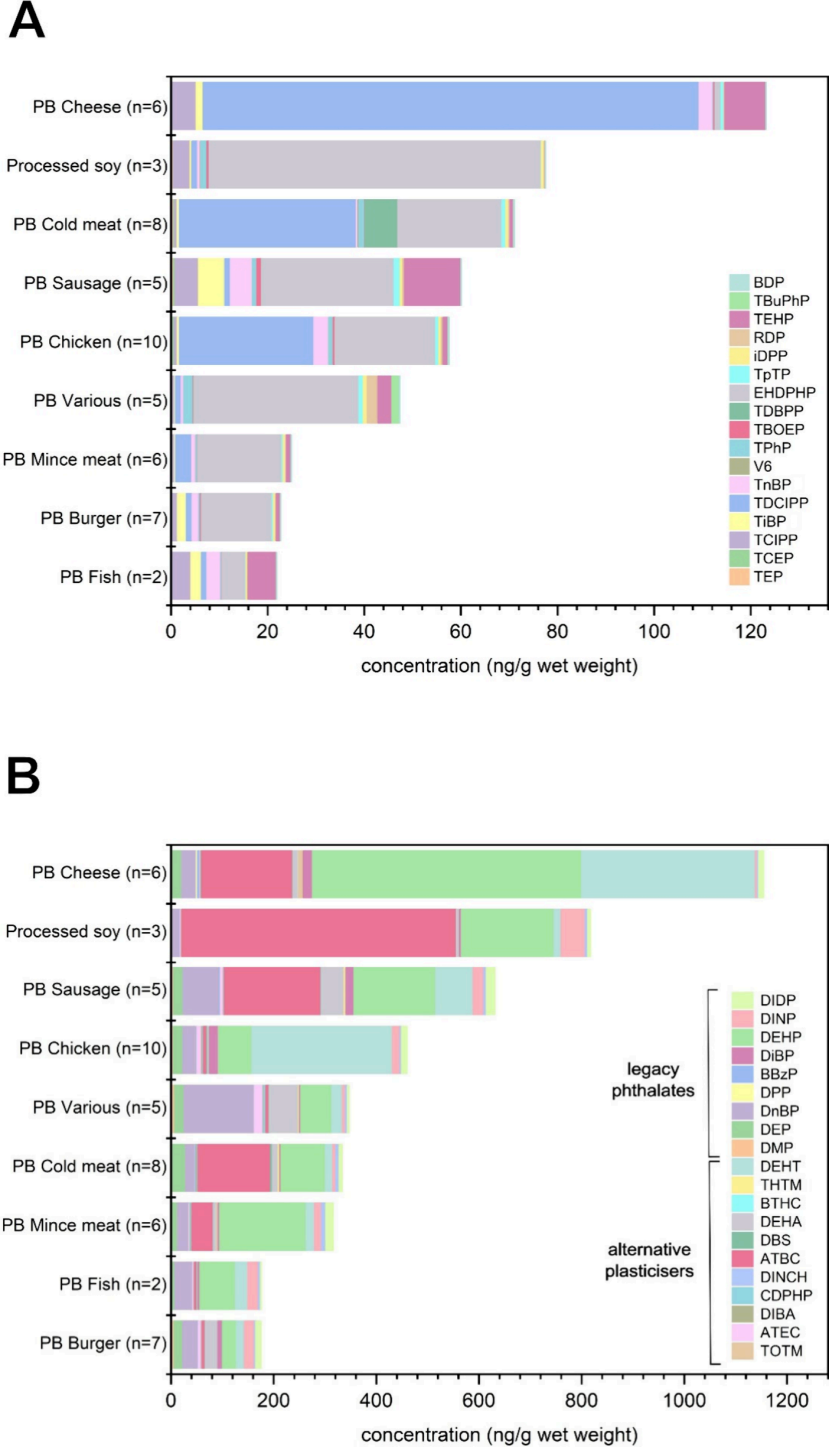
Average concentrations (ng/g ww) of (A) individual organophosphorus flame retardants (PFRs) and (B) legacy phthalates (LPs) and alternative plasticizers (APs) per plant-based (PB) food category (total NPBFs samples: *n* = 52) and contribution of individual compounds to the overall contamination.

Among PFRs, tris(2-ethylhexyl) phosphate (TEHP) had the highest detection frequency (DF, 81%) in NPBFs, followed by 2-ethylhexyl diphenyl phosphate (EHDPHP, 79%) (Table S5). Tris(1,3-dichloro-isopropyl) phosphate (TDCIPP) had the highest individual concentration (587 ng/g ww) in a PB cheese-alternative sample (PBP-10), which also had the highest total sum of PFRs (Table S6 and Figure S2A). Similarly, high concentrations of TDCIPP were measured in a PB chicken-alternative sample (PBP-16; 267 ng/g ww). Among the different PB food categories, PB cheese-alternatives were the most contaminated group (Table 1), with TDCIPP being the highest contributor to the sum (Figure 2A). This was followed by processed soy samples, which was mostly attributed to the concentrations of EHDPHP. TDCIPP (24 ng/g ww) and EHDPHP (22 ng/g ww) were also the compounds with the overall highest total average concentrations (Table S5). While EHDPHP is approved for use in food packaging and might originate from there,^17^ other PFRs such as TPhP are commonly used in lubricants^17^ and could have therefore been introduced through leaching from machinery during the production process.

**Table 1:** Total Average Concentrations (ng/g ww) and Standard Deviation (SD) of Organophosphorus Flame Retardants (PFRs), Legacy Phthalates (LPs), Alternative Plasticizers (APs), and Total Plasticizers (LPs+APs) per Plant-Based (PB) Food Category^a^

	∑PFRs		∑LPs		∑APs		∑total plasticizers	
	average	SD	average	SD	average	SD	average	SD
PB Burger (*n* = 7)	23	34	120	42	55	40	176	58
PB Cheese (*n* = 6)	123	238	612	267	543	405	1155	485
PB Chicken (*n* = 10)	58	87	159	69	301	817	460	820
PB Fish (*n* = 2)	22	8.7	138	23	38	0.8	176	23
PB Mince (*n* = 6)	25	33	236	193	80	88	316	212
Processed soy (*n* = 3)	78	119	255	151	562	706	817	722
PB Sausage (*n* = 5)	60	35	308	249	323	431	631	498
PB Various (*n* = 5)	47	37	235	171	112	66	347	184
PB Cold meat (*n* = 8)	71	93	150	140	184	381	333	406

a Total NPBFs samples: n = 52.

Among LPs, diethyl phthalate (DEP) had the highest DF of 77% in NPBFs (Table S7). DEHP had the highest individual concentration in a PB cheese-alternative sample (PBP-10) (Table S8, Figure S2B), and PB cheese-alternatives also had the highest total sum of LPs (Table 1). DEHP was the major contributor to the total LP levels in PB cheese-alternatives (Figure 2B) and the LP with the highest overall average concentration (144 ng/g ww) (Table S7). Among APs, cresyl diphenyl phosphate (CDPHP) had the highest DF (88%) in NPBFs (Table S7), while DEHT had the highest individual concentration in a PB chicken-alternative sample (PBP-23; 2,599 ng/g ww) (Table S8, Figure S2B). Processed soy samples also had the highest total sum of APs, followed by PB cheese alternatives (Table 1). DEHT was the major contributor to the total AP levels in PB cheese-alternatives, while ATBC was the main compound found in processed soy samples (Figure 2B). DEHT (109 ng/g ww) and ATBC (99 ng/g ww) were also the compounds with the overall highest average concentrations among APs (Table S7). While DEHP^38^ and DEHT^39^ are commonly used as plasticizers in PVC and could therefore have been introduced through contact with PVC equipment, ATBC is approved as a food contact additive.^40^

Despite the increasing popularity and consumption of NPBFs in Western countries, data on chemical safety are still scarce. To the best of our knowledge, there are only three other studies including meat-alternatives in food contamination studies. Ding et al. measured levels of PFRs up to 2 ng/g ww in tofu samples from China (n = 4),^41^ and van Holderbeke et al. found levels of LPs reaching 21 ng/g ww in meat-alternatives from Belgium (n = 5).^42^ In both cases, these values are lower than those measured in the current study, which is more recent and includes a higher number of both targeted compounds and PB food groups. On the other hand, the study of den Ouden et al. found higher concentrations of sum PFRs (33 ng/g ww) and plasticizers (349 ng/g ww) in Swedish food composite samples of meat-alternatives.^43^ This might be because the Swedish study analyzed three meat-alternative food composite samples, while the current study had a sample size of 52 individual NPBF samples from three European countries and included multiple PB categories, leading to a more representative contamination pattern.

In the current study, large variations in the PFR and plasticizer levels and patterns were observed between the different NPBFs samples (Figures S2A, S2B). One possible reason for this could be the diversity of the ingredients and compositions of these samples, which can vary widely (Table S1), since the base protein and additional ingredients are selected based on the characteristics of the desired end-product.^44^ PB cheese-alternatives, which consist mostly of coconut oil as the base ingredient, were the most contaminated food group, with both PFRs (123 ng/g ww) and plasticizers (1155 ng/g ww) (Table 1). All analyzed PB cheese-alternative samples had a fat content of around 20% (range 19–22%), among the fattiest samples in the data set. Fats and oils have been shown to be among the food categories most prone to both phthalate^38^ and PFR^15^ contamination, due to their lipophilic characteristics.^17^ However, it should be noted that only a few statistically significant correlations were observed between fat content and contamination levels, mostly for plasticizers (Table S9). This could be partly explained by the narrow range of fat content included in this sample set compared to studies which include multiple different food categories. Nevertheless, these results are arguable and indicate that there might be other factors that have a bigger impact on the prominent abundance of plasticizers and PFRs in PB cheese-alternatives rather than the fat content. Compared with previous studies, the contamination levels of PB cheese-alternatives were comparable to concentrations measured in commonly consumed oils and fats for both PFRs^15^ and phthalates^42^ but higher than in animal-based cheese for PFRs.^15^ While coconut oil has not been included in those studies, the fact that PB cheese-alternatives are mostly composed of coconut oil gives an indication that this might be a possible source of contamination. Additionally, while DEHP was the main contributor to contamination in PB cheese-alternatives, it could not be detected in the only sample which was based on nuts instead of coconut oil (PBP-11, Table S8, Figure S2). DEHP could therefore have already been introduced during coconut oil production, for example through processing equipment (e.g., PVC material or leaking from hydraulic oils).^38,45^ However, individual ingredients were not tested in the scope of this study, but they could be included in the frame of future research to give better insights into the specific sources of contamination.

Processed soy products were the second most contaminated food category for both PFRs (78 ng/g ww) and plasticizers (817 ng/g ww) (Table 1). These samples are considered less processed than other NPBFs, due to the lack of additive ingredients and processing steps that are usually involved to mimic the texture or taste of meat (Figure S1) and mostly consist of only a few ingredients (typically soy). The relatively high contamination of these samples was mostly attributed to the PFR EHDPHP and the AP ATBC (Figures 2A, 2B). Due to their use in plastic FCMs, the contamination of processed soy with EHDPHP and ATBC could have resulted from migration from FCMs, as previously suggested.^40,46^ Therefore, the levels of PFRs and plasticizers were also measured in the corresponding FCM to investigate their possible migration into the food.

### 3.2 PFRs, LPs, and APs in Food Contact Materials

**Figure 3:**
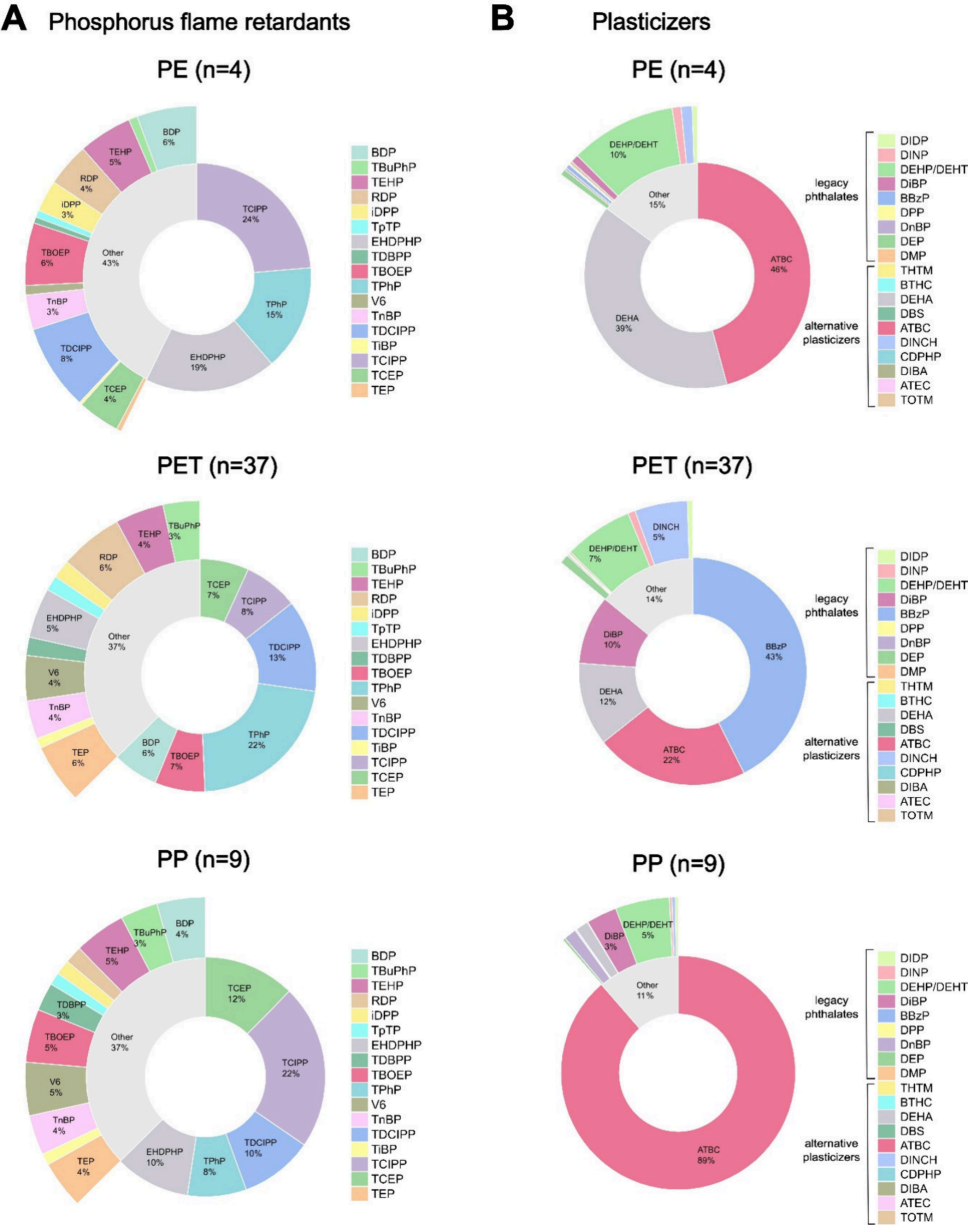
Relative % contribution of individual (A) organophosphorus flame retardants (PFRs) and (B) legacy phthalates (LPs) and alternative plasticizers (APs) to the overall occurrence in food contact materials (FCMs).

In the FCMs, TEHP and resorcinol bis(diphenyl phosphate) (RDP) had the highest DF (83%) among PFRs (Table S10). Generally, PFR concentrations were lower in FCM than in NPBFs, and tris(chloro-2-propyl) phosphate (TCIPP) (18 ng/g plastic) and TDCIPP (19 ng/g plastic) had the highest average concentrations (Table S10). TCIPP also had the highest individual concentration (234 ng/g plastic) in a mixed polypropylene (PP)/polyethylene (PE) sample (FCM-34, tightly wrapping a processed soy sample) (Table S11). Total PFRs ranged from 176 to 530 ng/g plastic in PE, 20–442 ng/g plastic in polyethylene terephthalate (PET), and 22–643 ng/g plastic in PP. The pattern of PFR contamination varied greatly between the type of plastic in FCM (Figure 3A); TCIPP was the most prevalent compound in PE and PP; in PET, the most prevalent compound was TPhP. Since some PFRs are also used as plastic additives in FCM and to investigate possible migration of the compounds, correlations between NPBF and FCM across food category and base ingredient were also investigated. While previous studies suggested possible migration of certain PFRs (such as EHDPHP) from the FCM to food, statistical analysis revealed only a few relevant correlations especially for TPhP (r = 0.88) and TBuPhP (r = 0.80) within certain food categories (Figure S3A). Diversely, no or low correlations were observed between NPBFs and FCMs for PFRs regarding the base ingredient (Figure S3C).

Among LPs, diphenyl phthalate (DPP) had the highest DF of 72%, while butyl benzyl phthalate (BBzP) had the highest total average concentration (1,742 ng/g plastic) (Table S12). Among APs, 1,2-cyclohexane dicarboxylic acid diisononyl ester (DINCH) was the most detected compound in FCMs, with a DF of 97% (Table S12). ATBC had the highest individual AP concentration (227,900 ng/g plastic) in a PP/PE sample (FCM-34) (Table S13) and also the highest total average concentration (7,170 ng/g plastic) (Table S12). Total plasticizers ranged from 8,460 to 54,100 ng/g plastic in PE samples, 271–112,865 ng/g plastic in PET samples, and 286–253,720 ng/g plastic in PP samples. Generally, APs contributed more to overall plasticizer contamination than LPs (ratio 6:1), and the patterns of contamination varied greatly between the different FCMs (Figure 3B). ATBC was the most prevalent AP in the PE and PP samples. In PET samples, BBzP was the most prevalent compound, followed by ATBC. The correlations between NPBF and FCM across food categories showed moderate correlations for ATEC, CDPHP, DEHA, DINCH, DEHP, and DEHT, while strong correlations were observed for DiBP (r = 0.84) and TOTM (r = 1.00) (Figure S3B). The correlations between NPBF and FCM based on base-ingredient showed that samples based on legumes had a moderate correlation (r = 0.60) for TOTM (Figure S3D). Additionally, DEP showed a high correlation between PB chicken-alternatives and their FCM, while DMP had high correlations in PB sausage-alternatives and DPP in PB cheese-alternatives, suggesting that migration of these compounds from the FCM to certain NPBFs could have occurred. Considering that these LPs are restricted for their use in FCMs due to their toxic properties (Da Costa et al., 2023), these results show the need for further monitoring of additives in FCM and their potential migration to different foods.

For the other NPBFs, the lack of correlation between concentrations of PFRs and plasticizers and their levels in FCMs can suggest that (i) a limited contact between NPBFs and their FCMs occurred, or (ii) that potential migration might simultaneously depend on various factors, including the polymer material or the food type,^25^ or (iii) that the observed contamination might have had a different source – including industrial processing. This latter option has been already shown in previous studies, where the degree of processing has been associated with chemical contamination.^15,16,38,47^ It has been suggested that both PFRs and plasticizers can be introduced into foodstuff during the production process, due to the use of processing equipment (such as PVC tubing or conveyor belts) made from materials which can contain those additives.^19,38,42,48,49^ A study by Fierens et al. monitoring phthalate contamination during the production process of milk indicated that contact materials used during the industrial processing were major contributors to the contamination in milk.^48^ Given the fact that most NPBFs are categorized as UPFs^50^ and therefore undergo multiple processing steps (Figure S1), it is suggested that industrial processing can be a likely source of contamination in these samples. Additionally, it has been shown that certain plasticizers can be found in PVC gloves used during food handling and can migrate into the food.^51,52^ Another possible source might also be the use of contaminated ingredients.^42^ Considering that most NPBFs consist of a large variety of −often already processed – ingredients (e.g., emulsifiers or thickening agents), these might also introduce chemical contaminants into the end-product. The distinctions in ingredients between different products as well as the diverse processing techniques that are used to manufacture NPBFs might also account for the variation in the contamination levels and patterns. It is therefore likely that the detected concentrations of contaminants in NPBFs can be attributed to contamination from multiple sources along the food supply and processing chain. This might also be a possible explanation for the high abundance of EHDPHP in NPBF samples, which was not prominent in FCM. It is for example possible that EHDPHP contamination was introduced through contact with PVC equipment during production or food packaging used during other steps of the manufacturing.^17^ This could even apply to processed soy samples, which also showed high EHDPHP concentrations.

While the production of processed soy samples (i.e., tofu, tempeh, and dry soy) does not incorporate the same extensive processing procedures as the majority of the other NPBF samples, such products still undergo manufacturing steps (such as grinding or heating of soybeans),^53^ which could introduce contamination. Nevertheless, the relatively high contamination levels for processed soy products (mostly attributed to EHDPHP and ATBC) were somewhat surprising. These products have among the highest soy content in the sample set, and while there is no information available on contamination in unprocessed soy, it can be hypothesized that the soy content might play a role in the case of the processed soy products. This also highlights the need for studies investigating the individual ingredients, as well as the end-product to elucidate contamination sources and factors.

### 3.3 Dietary Exposure Risk Assessment

**Figure 4:**
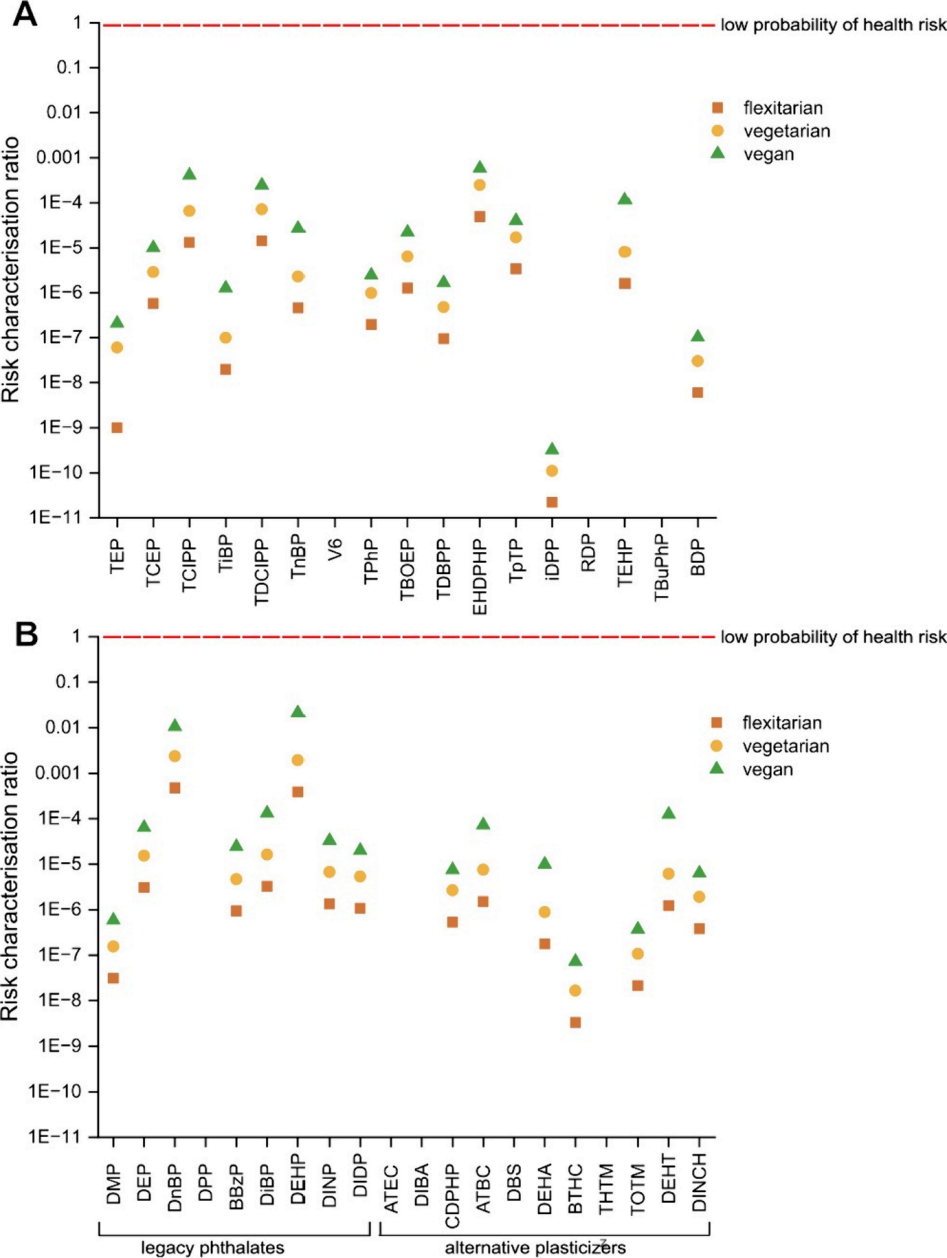
Dietary risk characterization of (A) organophosphorus flame retardants (PFRs) and (B) legacy phthalate (LPs) and alternative plasticizers (APs) in NPBFs. Risk characterization ratios (RCRs) were calculated for a flexitarian (10% substitution of meat consumption with PB meat-alternative), vegetarian (50% substitution of meat consumption with PB meat-alternative), and vegan scenario (100% substitution of meat, fish, and cheese consumption with corresponding NPBFs). The most conservative HBGVs were used to calculate RCRs for each compound in the three scenarios (Tables S14 and S15). RCRs below 1 indicate that the estimated exposure is unlikely to pose a significant health risk for the adult population.

The potential adverse health risks for humans associated with the ingestion of NPBFs were evaluated through a dietary exposure risk assessment. Due to the novelty of such products, there is currently a lack of consumption data regarding NPBFs. Therefore, the EDI was calculated based on three different assumptive scenarios: flexitarian diet, vegetarian diet, and vegan diet (Figure 1). For sum PFRs, the estimated daily intake (EDI) was 2.7 ng/kg bw/day in the flexitarian scenario and 14 ng/kg bw/day in the vegetarian scenario (Table S14). The EDI in the vegan scenario reached 53 ng/kg bw/day, with PB meat alternatives being the major contributor (27 ng/kg bw/day). Calculated EDIs in the vegan scenario were still at least 1000 times lower than available HBGVs, and the risk characterization ratios were consistently below 1 (Figure 4A, Table S14), indicating that the estimated exposure is unlikely to pose a significant health risk for the adult population.

For total plasticizers, the EDI was 31 ng/kg bw/day in the flexitarian scenario (LPs 23 ng/g ww, APs 8 ng/g ww) and 154 ng/kg bw/day in the vegetarian scenario (LPs 113 ng/g ww, APs 42 ng/g ww) (Table S15). The EDI in the vegan scenario reached 1,571 ng/kg bw/day (LPs 961 ng/g ww, APs 610 ng/g ww), with PB cheese-alternatives contributing the most (1,169 ng/kg bw/day, ratio LP/AP 1:1). Generally, LPs had higher EDIs than APs in all scenarios. Also in this case, calculated EDIs for the vegan scenario were still at least 100 times lower than the HBGVs for all compounds (Figure 4B, Table S15), suggesting that health risks for the adult population via NPBF ingestion are limited.

**Figure 5:**
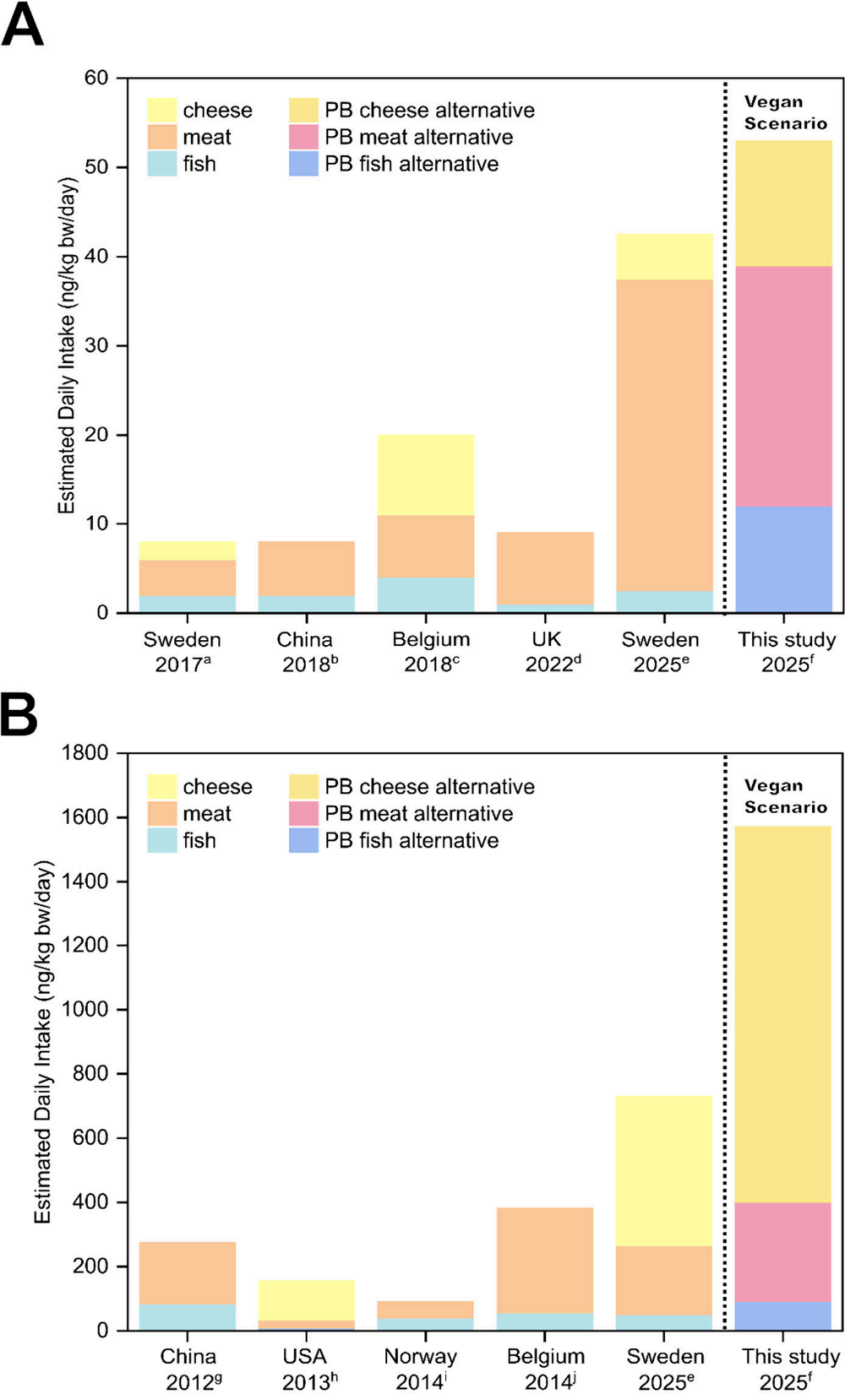
Estimated daily intake (EDI, ng/kg bw/day) of (A) organophosphorus flame retardants (PFRs) and (B) legacy phthalates (LPs) and alternative plasticizers (APs). EDIs for animal products (fish, meat, and cheese) from other studies were compared to the EDI calculated in this study based on the contamination of PFRs and plasticizers in NPBFs. The EDI was calculated based on consumption data derived from Germany, and the animal products (fish, meat, and cheese) were substituted by the corresponding NPBFs. Based on that, a vegan scenario (100% replacement of ABFs) was calculated. ^a^ (Poma et al., 2017), ^b^ (Ding et al., 2018), ^c^ (Poma et al., 2018) ^d^ (Gbadamosi et al., 2022), ^e^ (den Ouden et al., 2025), ^f^ (this study), ^g^ (Guo et al., 2012), ^h^ (Schecter et al., 2013), ^i^ (Sakhi et al., 2014), ^j^ (Fierens et al., 2014).

Compared with other studies on ABPs, the EDI calculated for a vegan scenario was higher for both PFRs and plasticizers (Figures 5A, 5B). It should be noted, however, that there are differences in the targeted compounds between studies, and consumption habits might also differ between countries. While the health risks associated with the exposure to all compounds in the three scenarios can be considered adequately controlled for the adult population, the LPs DEHP and DnBP had RCR values above 0.01 in the vegan scenario (Figure 4B). It should be considered that EDIs were calculated for a vegan scenario in which meat, fish, and cheese were substituted, but other food groups were not included in this exposure assessment. Including additional food groups in the exposure risk assessment would likely increase the EDI and RCR values. Additionally, in this study, only the dietary pathway was considered, while overall exposure to these compounds can also occur via other routes, such as dust ingestion and inhalation or dermal contact.^19,38^ While diet is an important pathway for exposure to PFRs and plasticizers, other pathways that can also contribute were not included in this study; it can therefore be assumed that human exposure would increase if multiple pathways were considered, resulting in a higher risk. Eventually, a comprehensive exposure assessment could be performed in the future by including multiple relevant pathways and accounting for their contribution in the total aggregate exposure or by using a biomonitoring approach and reflecting on the contribution of each route in order to design mitigation strategies.

### 3.4 Strengths and Limitations

This was the first study investigating the occurrence of three important environmental contaminant classes in NPBFs and provided valuable and novel insights into their chemical food safety. This comprehensive assessment included a broad range of a representative selection of samples (n = 52) that were purchased in different European countries in the course of 2023. By its design, this study did not focus on certain PB food categories, such as PB milk-alternatives. However, based on the results, it would be recommended to investigate specific PB food categories. Additionally, the availability and range of products on the European market might have expanded since the time of purchase (2023).

Industrial food processing and FCMs have been identified as potential sources of PFR and plasticizer contamination in NPBFs. Unfortunately, the impact of certain processing methods on contamination levels remained unknown, as the testing of individual processing steps was not possible in this study.

Additionally, the data regarding consumption of NPBFs (dietary patterns, quantity, and frequency) are currently still lacking due to the novelty of these products, and this exposure and risk assessment can therefore only be seen as a first precautionary estimation; exposure via NPBF ingestion and possible health outcomes should be reevaluated in the future after sufficient applicable data has been made available. The broad range of compounds included in this study provided valuable first insights on the chemical food safety of NPBFs; however, an additional investigation of other contaminants which might accumulate in such products is recommended.

Further studies are therefore crucial to assess the food safety of NPBFs and should focus on (i) including a broad and representative selection of samples; (ii) expanding the analysis to other compound classes (e.g., compounds associated with industrial processing such as chlorinated paraffins or with plant ingredients such as pesticides); (iii) assessing specific sources of contamination by, for example, collecting samples during multiple stages of the production process or by testing individual ingredients; (iv) acquiring comprehensive consumption data on vegan and vegetarian diets, and finally, (v) combining data of multiple compound classes to perform a reliable safety and exposure assessment of such products. Eventually, NPBFs (for example, different PB meat- or milk-alternatives) should be included in food monitoring studies alongside their animal-based homologues, to have a more accurate estimation of dietary exposure.

In conclusion, these findings show several differences in contamination levels and patterns between PB food categories, with PB cheese-alternatives being the most contaminated group for both PFRs and plasticizers. Industrial food processing and the migration of chemicals from FCMs were identified among the possible sources of contamination of NPBFs. Generally, the NPBFs in this study showed higher contamination levels compared to their animal homologues, and a dietary exposure risk assessment revealed higher exposure in a conservative vegan diet scenario compared to other studies on ABFs. Given their increasing popularity and consumption rate, we recommend including NPBFs of different categories in future consumption surveys, market basket studies, and human risk assessments based on food ingestion. Finally, while this study showed that the adult population is not at immediate risk following the intake of such NPBFs, caution is advised when basing one’s diet entirely on these UPFs, and a direct replacement of all animal products solely with NPBFs is not recommended.

## Data Availability

Additional data can be made available upon request.

## Supporting Information Available

Additional data for this study, additional information on the samples, experimental details (LC/MS acquisition), materials and methods (information on chemicals and consumption data), individual PFR and plasticizer concentrations in NPBFs and FCMs, additional statistical information (descriptive statistics and correlations of NPBF samples), calculated exposure and risk assessments and an overview of the industrial processing steps for NPBFs (PDF)
