# Supplementary material for: Organophosphorus Flame Retardant, Phthalate, and Alternative Plasticizer Contamination in Novel Plant-Based Food: A Food Safety Investigation
Source: Environ Sci Technol. 2025 Mar 21;59(18):9209–20. doi: 10.1021/acs.est.4c11805 (PMC12080256; doi:10.1021/acs.est.4c11805)
Supplement: Supplementary file 1 — es4c11805_si_001.pdf [file es4c11805_si_001.pdf]

Organophosphorus flame retardant, phthalate and alternative plasticizer contamination in novel plant-based food: a food safety investigation

Alicia Macan Schönleben [a*], Fatima den Ouden [a], Shanshan Yin [a,b], Erik Fransen [c], Stijn Bosschaerts [a], Mirjana Andjelkovic [d], Nayyer Rehman [e], Alexander L. N. van Nuijs [a], Adrian Covaci [a], Giulia Poma [a*]

[a] Toxicological Centre, University of Antwerp, Universiteitsplein 1, 2610 Wilrijk, Belgium

[b] Key Laboratory of Pollution Exposure and Health Intervention of Zhejiang Province, Interdisciplinary Research Academy (IRA), Zhejiang Shuren University, Hangzhou 310015, China

[c] Centre of Medical Genetics, University of Antwerp and Antwerp University Hospital, 2650 Edegem, Belgium; Centre for Oncological Research, University of Antwerp and Antwerp University Hospital, 2610 Wilrijk, Belgium

[d] Sciensano, Service Risk and Health Impact Assessment, Juliette Wytsmanstraat 14, 1050 Brussels, Belgium

[e] WRG Europe Ltd, 26-28 Southernhay East, EX1 1NS, Exeter, UK

* Corresponding authors:

Giulia Poma, Giulia.Poma@uantwerpen.be

Alicia Macan Schönleben, Alicia.MacanSchonelebn@uantwerpen.be

Summary: 30 Pages, 15 Tables, 3 Figures

Table of contents

Section 1. Description of novel plant-based foods. 3

Section 2. Analytical methods. 3

Table S 1. Sample-ID, food category, sample description, brand and purchase store codes, purchase country, base ingredient, packaging material and details, water and fat content of the individual NPBFs. 4

Table S 2. Details on purchased standards, chemical and materials. 6

Table S 3. List of targeted compounds (abbreviations and full names), accuracy (%) for NPBFs and food contact material (FCM), analytical instrument used, limits of quantification (LOQs) in ng/g wet weight for NPBF and ng/g plastic for FCM samples per analysed batches 7

Table S 4. Average consumption data of meat, fish and cheese in g/day per person. 9

Table S 5. Descriptive statistics for PFR contamination in ng/g wet weight (ww) per novel plant-based food (NPBF) category. 10

Table S 6. Individual PFR concentrations in ng/g ww in NPBF samples. 12

Table S 7. Descriptive statistics for LP and AP contamination in ng/g ww per NPBF category. .. 14

Table S 8. Individual LP and AP concentrations in ng/g ww in NPBF samples. 16

Table S 9. Correlation coefficients and p-values for correlations between individual and total PFR, LP and AP concentrations in NPBFs and fat content of the samples. 18

Table S 10. Descriptive statistics in ng/g plastic and detection frequencies of PFRs in FCM samples. 19

Table S 11. Individual PFR concentrations in ng/g plastic in FCM samples. 20

Table S 12. Descriptive statitistics in ng/g plastic and detection frequencies of LP and AP contamination in FCM samples. 22

Table S 13. Individual LP and AP concentrations in ng/g plastic in FCM samples. 23

Table S 14. Exposure and risk assessment for individual PFRs. 25

Table S 15. Exposure and risk assessment for individual plasticizers. 26

Figure S 1. Overview of industrial process steps for the production of novel plant-based foods. Created 27

Figure S 2. Individual (A) PFR and (B) plasticizer contamination in ng/g wet weight per NPBF sample and contribution of compounds to the overall contamination. 28

Figure S 3. Correlations between (A, B) FCM and NPBF contamination for individual PFRs and plasticizers according to food category and (C, D) base ingredient. 29

References 30

Section 1. Description of novel plant-based foods.

Due to a lack of commonly agreed upon terminologies, this study uses terminologies as defined by Espinosa et al. [1]. Novel plant-based foods (NPBFs) therefore refer to plant-based (i.e. vegan and vegetarian) alternatives which intend to replace animal products (such as meat or cheese) without using animal-originated ingredients. To manufacture products with the properties of animal-based foods (ABFs) without in fact using any animal ingredients multiple food processing techniques (such as high-pressure extrusion) are required, in which the proteins are extracted from the whole food (such as soybeans) and combined with other additive ingredients (such as emulsifiers or flavouring agents) (Figure S1) [2].

Due to the use of additives as well as the extensive manufacturing process involved in the production of NPBFs, they are considered ultra-processed food (UPF) according to the NOVA classification system [3,4]. This system places food in 4 categories; unprocessed or minimally processed food (group 1), processed culinary ingredients (group 2), processed food (group 3) and UPFs (group 4). This latter group includes processes in which the whole food is first dissembled into chemical components and then afterwards reassembled and combined with additives – in the case of NPBFs to mimic the properties of ABFs. Some characteristic ultra-processed ingredients in NPBFs include protein isolates or methyl cellulose, which can be found in most products as a stabilizer. While the natural tofu, tempeh and dry soy samples in this study mostly did not contain such additive ingredients and can therefore not be placed as UPFs, they are still considered processed food under the NOVA classification system due to the manufacturing process they need to undergo to reach the end-product [5].

Section 2. Analytical methods.

Target analyses were performed using previously described validated methods [6–8]. The LC analysis was carried out using an Agilent Infinity 1200 liquid-chromatography system coupled to an Agilent 6410 Triple Quadrupole mass spectrometer equipped with an electrospray ionization source (Agilent Technologies) using positive ionization mode. Chromatographic separation was achieved using a Kinetex Biphenyl column (2.1 mm x 100 mm, 2.6 µm, Phenomenex) with water (A) and methanol (B) as mobile phases, both containing 5 mM ammonium formate. The acquisition was carried out using dynamic multiple reaction monitoring mode in positive electrospray ionization mode. The sheath gas temperature was set to 350 °C at a flow of 10 L/min and the nebulizer pressure was set at 40 psi. Capillary and nozzle voltage were set to 4000 V and 500 V, respectively. The injection volume was 5 µL and the column temperature was 40 °C.

For the GC analysis of DEHP and DEHT, an Agilent gas-chromatography was used coupled to an Agilent 5973 MA operated in electron ionization mode. Chromatographic separation was achieved using a GC HT-8 column (25 m x 0.22 mm, 0.25 µm) with electronic pressure control and a programmable-temperature vaporizer inlet. The injection temperature was set at 90 °C, hold time 0.04 min, ramped at 700 °C/min to 300 °C, hold time 25 min. The injection volume was set to 1 µL with a pressure of 14.36 psi until 1.25 min and purge flow to split vent of 50 mL/min after 1.25 min. Helium was used as a carrier gas with a flow rate of 1 mL/min until 28 min and then increased to 1.5 mL/min. The acquisition was carried out using selected ion monitoring mode with 2 characteristic ions acquired for each analyte and the IS.

Table S 1. Sample-ID, food category, sample description, brand and purchase store codes, purchase country, base ingredient, packaging material and details, water and fat content of the individual NPBFs.

Sample-ID	Food category	Description	Brand code	Store code	Country [a]	Base ingredient	Packaging material [b]	Packaging details	Packaging area (cm^2) [c]	Water content (%)	Fat content [b] (%)
**PBP-01**	PB Burger	Burger	1	A	Belgium	Legumes	PP	Plastic tray	1.5	59	19
**PBP-02**	PB Burger	Burger	2	A	Belgium	Soy	PET	Plastic tray	1.6	59	7.5
**PBP-03**	PB Burger	Burger	5	A	Belgium	Soy	PET	Plastic tray	1.9	53	8.2
**PBP-04**	PB Burger	Burger	6	A	Belgium	Legumes	PET	Plastic tray	2.0	53	10
**PBP-05**	PB Burger	Burger	11	D	Germany	Soy	PET	Plastic tray	1.8	61	7.7
**PBP-06**	PB Burger	Burger	16	E	United Kingdom	Legumes	PET	Plastic tray	1.4	57	17
**PBP-07**	PB Burger	Burger	17	F	Germany	Legumes	n.a.	Plastic tray	5.4	55	10
**PBP-08**	PB Cheese	Cheese	6	A	Belgium	Oil	PET	Plastic tray	1.5	52	22
**PBP-09**	PB Cheese	Cheese	8	B	Belgium	Oil	PET	Plastic tray	1.7	54	21
**PBP-10**	PB Cheese	Cheese	12	A	Belgium	Oil	PET	Plastic tray	1.2	51	20
**PBP-11**	PB Cheese	Cheese	13	C	Belgium	Nuts and seeds	PET	Plastic tray	1.7	54	19
**PBP-12**	PB Cheese	Cheese	14	F	Germany	Oil	PET	Plastic tray	1.6	45	21
**PBP-13**	PB Cheese	Cheese	17	F	Germany	Oil	PET	Plastic tray	1.5	46	21
**PBP-14**	PB Chicken	Schnitzel	1	A	Belgium	Legumes	PP	Plastic tray	1.5	44	14
**PBP-15**	PB Chicken	Schnitzel	2	A	Belgium	Soy	PET	Plastic tray	1.6	37	7.8
**PBP-16**	PB Chicken	Tender chicken	3	A	Belgium	Soy	PP	Plastic tray	1.3	46	11
**PBP-17**	PB Chicken	Chicken	4	A	Belgium	Mycoprotein	PP	Plastic tray	1.4	69	2.6
**PBP-18**	PB Chicken	Schnitzel	5	F	Germany	Soy	PET	Plastic tray	1.9	47	18
**PBP-19**	PB Chicken	Chicken	7	A	Belgium	Soy	PET	Plastic tray	1.7	64	1.8
**PBP-20**	PB Chicken	Nuggets	10	C	Belgium	Soy	PET	Plastic tray	1.4	47	8.0
**PBP-21**	PB Chicken	Schnitzel	14	F	Germany	Wheat	PET	Plastic tray	1.7	47	16
**PBP-22**	PB Chicken	Schnitzel	15	F	Germany	Soy	PET	Plastic tray	1.7	55	10
**PBP-23**	PB Chicken	Chicken bites	17	E	United Kingdom	Wheat	AL/PE	Plastic bag	5.5	31	21
**PBP-24**	PB Fish	Fish sticks	6	A	Belgium	Legumes	PET	Plastic tray	2.0	50	14
**PBP-25**	PB Fish	Fish	11	D	Germany	Soy	PET	Plastic tray	1.8	58	4.4
**PBP-26**	PB Mince	Mince Meat	1	A	Belgium	Legumes	PP	Plastic tray	1.5	54	17
**PBP-27**	PB Mince	Mince Meat	5	A	Belgium	Soy	PET	Plastic tray	1.9	57	0.5

**PBP-28**	PB Mince	Mince Meat	6	A	Belgium	Legumes	PET	Plastic tray	2.0	55	9.4
**PBP-29**	PB Mince	Mince Meat	11	D	Germany	Soy	PET	Plastic tray	1.8	57	9.0
**PBP-30**	PB Mince	Mince Meat	14	F	Germany	Legumes	PP	Plastic tray	3.0	80	18
**PBP-31**	PB Mince	Mince Meat	15	F	Germany	Soy	PET	Plastic tray	1.7	65	9.3
**PBP-32**	Processed soy	Tempeh	6	A	Belgium	Soy	n.a.	Wrapped	11	64	7.5
**PBP-33**	Processed soy	Tofu	8	B	Belgium	Soy	n.a.	Vacuum	7.5	74	5.9
**PBP-34**	Processed soy	Soy (dry)	14	F	Germany	Soy	PP/PE	Plastic bag	7.8	0	8.3
**PBP-35**	PB Sausage	Sausage	1	A	Belgium	Legumes	PP	Plastic tray	1.5	53	16
**PBP-36**	PB Sausage	Sausage	2	A	Belgium	Soy	PP	Plastic tray	1.6	56	10
**PBP-37**	PB Sausage	Sausage	6	A	Belgium	Legumes	PET	Plastic tray	2.0	59	8.2
**PBP-38**	PB Sausage	Sausage	18	E	United Kingdom	Legumes	PET	Plastic tray	1.8	58	12
**PBP-39**	PB Sausage	Sausage	19	G	Belgium	Wheat	n.a.	Vacuum	7.5	51	20
**PBP-40**	PB Various	Drumsticks	3	A	Belgium	Soy	PET	Plastic tray	1.4	48	8.6
**PBP-41**	PB Various	Beet Burger	6	A	Belgium	Vegetables	PET	Plastic tray	2.0	73	0.2
**PBP-42**	PB Various	Salami	14	F	Germany	Legumes	PET	Plastic tray	1.6	58	19
**PBP-43**	PB Various	Meatball	15	F	Germany	Wheat	PET	Plastic tray	1.7	56	10
**PBP-44**	PB Various	Salami	17	E	United Kingdom	Wheat	AL/PE	Plastic bag	5.5	36	23
**PBP-45**	PB Cold meat	Cold meat	2	A	Belgium	Milk	PET	Plastic tray	2.3	58	14
**PBP-46**	PB Cold meat	Cold meat	4	A	Belgium	Mycoprotein	PET	Plastic tray	1.9	68	3.1
**PBP-47**	PB Cold meat	Cold meat	5	A	Belgium	Soy	PET	Plastic tray	1.9	64	5.0
**PBP-48**	PB Cold meat	Cold meat	6	A	Belgium	Legumes	PET	Plastic tray	2.4	64	11
**PBP-49**	PB Cold meat	Cold meat	10	C	Belgium	Milk	PET	Plastic tray	2.0	61	16
**PBP-50**	PB Cold meat	Cold meat	11	D	Germany	Legumes	PET	Plastic tray	1.9	61	17
**PBP-51**	PB Cold meat	Cold meat	14	F	Germany	Legumes	PE/PET	Wrapped	11	66	11
**PBP-52**	PB Cold meat	Cold meat	15	F	Germany	Nuts and seeds	PET	Plastic tray	1.3	68	9.2

[a] Country refers to the country each product was purchased. [b] Fat content and packaging material information was acquired by vendor; polyethylene (PE), polypropylene (PP), polyethylene terephthalate (PET), aluminium (AL). [c] Packaging area refers to the area in cm^2 that corresponds to 50 mg of analysed plastic. N.a.: not available

Table S 2. Details on purchased standards, chemical and materials.

Item	Supplier
**Individual standards**	
TDBPP, V6, RDP, iDDP, DMP, DEP, DNBP, DIBP, BBzP, DEHP, DPP, DIBA, ATEC, DBS, ATBC, DEHA, CDPHP, BTHC, DEHT, THTM, TOTM, DINCH, DINP, DIDP, DBzP-d4	AccuStandard (St. Louis, USA)
DEHP-d4, DNBP-d4	Sigma-Aldrich (St. Louis, USA)
TIBP, TNBP, TDCIPP, TCIPP, TEHP, TCEP, TOTP, TPTP, TMTP, EHDPHP, TEP, TPHP, TBOEP, TBuPH	Chiron AS (Trondheim, Norway)
TAP (Recovery standard)	TCI Europe (Zwijndrecht, Belgium)
**Custom-synthesised standards**	
TPHP-d15, TBOEP-d6, TCEP-d12 and TDCIPP-d15	Dr. Vladimir Belov, Max Planck Institute for Biophysical Chemistry (Göttingen, Germany)
Florisil 2 cc Vac cartridge (500 mg)	Waters (Milford, USA)
Sodium chloride (NaCl)	Sigma-Aldrich (St. Louis, USA)
Sorbents C18 and primary-secondary amine (PSA)	Supelco (Bellefonte, USA)
Centrifugal filters (modified nylon, 0.2 µm)	VWR International (Leuven, Belgium)
Hexane	Acros Organics (Belgium)
Acetonitrile	Merck (Darmstadt, Germany)
Dichloromethane	Merck (Darmstadt, Germany)
Acetone	Merck (Darmstadt, Germany)
Toluene	Merck (Darmstadt, Germany)
Methanol	Merck (Darmstadt, Germany)
LC-grade ultrapure water	PURELAB Flex System (Tienen, Belgium)

Table S 3. List of targeted compounds (abbreviations and full names), accuracy (%) for NPBFs and food contact material (FCM), analytical instrument used, limits of quantification (LOQs) in ng/g wet weight for NPBF and ng/g plastic for FCM samples per analysed batches.

Compound	Abbreviation	Accuracy (%)		MRM transition	Instrument	LOQ NPBF			LOQ FCM	
**Phosphorus flame retardants**		**PBP**	**FCM**			**batch 1**	**batch 2**	**batch 3**	**batch**	**batch 2**
2-ethylhexyl diphenyl phosphate	EHDPHP	153	60	363.2->251.0	LC/MS	0.5	1	-	5	5
Tris (2-butoxyethyl) phosphate	TBOEP	89	135	399.3->299.2	LC/MS	0.3	2	-	0.5	0.5
Tri-iso-butyl phosphate	TiBP	100	116	267.2->99.0	LC/MS	1	1	-	10	10
Tri-N-butyl phosphate	TnBP	119	121	267.2->99.0	LC/MS	0.5	2	-	7	7
Tris(4-tert-butylphenyl) phosphate	TBuPhP	123	161	495.2->327.0	LC/MS	0.15	0.02	-	0.5	0.5
Tris (2-chloroethyl) phosphate	TCEP	96	182	285.0->63.0	LC/MS	0.5	0.5	-	1.5	1.5
Tris(2-chloro-2-propyl) phosphate	TCIPP	114	134	327.1->99.0	LC/MS	5	5	-	30	30
Tris(1,3-dichloro-2-propyl) phosphate	TDCIPP	109	184	432.9->99.0	LC/MS	10	10	-	20	20
Tris(2-ethylhexyl) phosphate	TEHP	130	131	435.3->99.0	LC/MS	0.15	0.05	-	0.5	0.5
Triethyl phosphate	TEP	45	126	183.1->99.0	LC/MS	1	0.5	-	5	5
Triphenyl phosphate	TPhP	105	199	327.0->77.0	LC/MS	0.3	0.3	-	3	3
Tri-p-cresyl phosphate	TpTP	112	199	369.2->165.0	LC/MS	0.15	0.02	-	3	3
Bisphenol A - bis(diphenyl phosphate)	BDP	75	205	710.1->367.0	LC/MS	0.1	0.02	-	5	5
Isodecyl diphenyl phosphate	iDPP	83	61	391.2->251.0	LC/MS	0.3	0.1	-	0.1	0.1
Resorcinol bis(diphenyl phosphate)	RDP	86	143	592.1->575.0	LC/MS	0.15	0.8	-	0.1	0.1
Tris (2,3-dibromopropyl) phosphate	TDBPP	54	89	698.5->99.0	LC/MS	0.01	0.01	-	0.01	0.01
2,2-bis(chloromethyl)-propane-1,3-diyltetrakis(2-chloroethyl) biphosphate	V6	39	166	582.9->360.9	LC/MS	0.1	0.01	-	0.5	0.5
**Legacy phthalates**										
Benzyl butyl phthalate	BBzP	119	80	313.2->148.9	LC/MS	0.5	0.5	3	3	10
Bis(2-ethylhexyl) phthalate	DEHP	109	152	-	GC/MS	120	300	-	350	350
Diethyl phthalate	DEP	91	127	223.1->148.9	LC/MS	2	2	5	15	15
Di iso decyl phthalate	DIDP	-	-	395.3->153.0	LC/MS	5	5	10	150	150
Di iso nonyl phthalate	DINP	-	-	419.3->71.2	LC/MS	5	5	10	50	30
Dimethyl phthalate	DMP	58	90	195.1->163.0	LC/MS	1	1	2	5	5
Di-n-butyl phthalate	DnBP	115	102	279.2->149.0	LC/MS	20	180	70	50	50
Diphenyl phthalate	DPP	119	100	319.2->225.0	LC/MS	0.15	0.15	0.03	0.5	0.5

**Alternative plasticizers**										
Acetyltributyl citrate	ATBC	199	71	403.3->129.0	LC/MS	50	30	50	250	250
Acetyltriethyl citrate	ATEC	138	78	319.0->157.0	LC/MS	3	3	0.01	1	0.05
Butyryl trihexyl citrate	BTHC	128	108	515.3->129.0	LC/MS	0.01	0.1	0.5	2	0.5
Cresyl diphenyl phosphate	CDPHP	151	64	341.1->91.9	LC/MS	0.15	0.15	0.15	0.1	1
Dibutyl sebacate	DBS	124	52	315.2->185.0	LC/MS	0.5	3	0.5	0.5	1
Diethylhexyl adipate	DEHA	119	127	371.3->129.1	LC/MS	10	10	100	30	30
Bis (2-ethylhexyl) terephthalate	DEHT	104	-	-	GC/MS	70	200	-	-	-
Di-iso-butyl adipate	DIBA	98	64	259.2->129.0	LC/MS	0.15	0.15	0.5	1	2
1,2- Cyclohexane dicarboxylic acid diisononyl	DINCH	-	-	526.3->155.0	LC/MS	1	20	20	2	2
Tri-n-hexyl trimellitate	THTM	108	57	436.3->277.0	LC/MS	0.01	0.01	0.02	1	0.1
Tris(2-ethylhexyl) trimellitate	TOTM	74	34	547.5->305.2	LC/MS	1	1	1	10	30

Table S 4. Average consumption data of meat, fish and cheese in g/day per person.

	Meat consumption	Fish consumption	Cheese consumption
**Belgium, *2014*[a]**	155	28	40
**Germany, *2023*[b]**	141	37	67

[a] European Food Safety Authority database (EFSA);
[b] German Federal Ministry of Food and Agriculture (BMEL)

Table S 5. Descriptive statistics for PFR contamination in ng/g wet weight (ww) per novel plant-based food (NPBF) category.

		TEP	TCEP	TCIPP	TiBP	TDCIPP	TnBP	V6	TPhP	TBOEP	TDBPP	EHDPHP	TpTP	iDPP	RDP	TEHP	TBuPhP	BDP	∑PFRs
**DF (%)**		12	4	13	21	12	25	21	58	10	8	79	71	58	35	81	27	54	
**PB Burger (n=7)**																			
	mean	0.2	0.02	1.2	1.8	1.2	1.4	0.01	0.3	0.2	0.01	15	0.3	0.4	0.1	0.9	0.02	0.04	**23**
	median	0.1	0.02	0.7	0.2	1.2	0.5	0.01	0.2	0.1	0.01	1.4	0.1	0.2	0.0	0.3	0.01	0.05	**5.0**
	SD	0.3	0.0	1.3	3.3	0.0	2.1	0.01	0.2	0.3	0.0	33	0.4	0.6	0.2	1.5	0.02	0.03	**34**
	min	0.1	0.02	0.7	0.2	1.2	0.1	0.002	0.2	0.03	0.01	0.4	0.01	0.1	0.03	0.04	0.01	0.01	**3.0**
	max	0.8	0.02	4.2	9.0	1.2	5.9	0.03	0.6	0.8	0.01	90	0.9	1.6	0.6	4.2	0.04	0.1	**120**
**PB Cheese (n=6)**																			
	mean	0.1	0.02	5.1	1.5	103	2.9	0.01	0.2	0.3	0.01	1.2	0.6	0.1	0.03	8.5	0.02	0.05	**123**
	median	0.1	0.02	0.7	1.2	1.2	3.6	0.002	0.2	0.1	0.01	0.8	0.1	0.1	0.03	7.0	0.01	0.05	**15**
	SD	0.03	0.0	7.2	1.4	238	1.9	0.01	0.0	0.4	0.0	0.8	1.1	0.1	0.002	9.8	0.02	0.03	**238**
	min	0.1	0.02	0.7	0.2	1.2	0.5	0.002	0.2	0.03	0.01	0.4	0.0	0.1	0.03	0.1	0.01	0.01	**3.5**
	max	0.1	0.02	17.7	3.3	587	5.3	0.02	0.2	1.2	0.01	2.4	2.7	0.2	0.03	26.4	0.04	0.1	**647**
**PB Chicken (n=10)**																			
	mean	0.2	0.5	0.7	0.4	28	3.1	0.02	0.9	0.4	0.01	21	0.7	0.5	0.3	1.0	0.1	0.2	**58**
	median	0.1	0.02	0.7	0.2	1.2	0.5	0.002	0.8	0.1	0.01	17	0.4	0.2	0.2	1.0	0.04	0.1	**22**
	SD	0.3	1.5	0.00	0.5	84	8.1	0.03	0.9	1.0	0.0	21	0.7	0.6	0.5	0.7	0.2	0.3	**87**
	min	0.1	0.0	0.7	0.2	1.2	0.1	0.002	0.2	0.03	0.01	0.4	0.04	0.1	0.03	0.1	0.01	0.01	**3.1**
	max	1.2	4.8	0.7	1.4	267	26	0.1	3.2	3.1	0.01	60	2.2	1.9	1.7	2.1	0.6	1.0	**377**
**PB Fish (n=2)**																			
	mean	0.1	0.02	4.0	2.2	1.2	2.8	0.01	0.2	0.1	0.01	4.9	0.1	0.4	0.03	5.9	0.01	0.05	**22**
	median	0.1	0.02	4.0	2.2	1.2	2.8	0.01	0.2	0.1	0.01	4.9	0.1	0.4	0.03	5.9	0.01	0.05	**22**
	SD	0.0	0.0	4.8	2.8	0.0	3.3	0.01	0.0	0.0	0.0	5.8	0.1	0.4	0.0	0.7	0.0	0.1	**9**
	min	0.1	0.02	0.7	0.2	1.2	0.5	0.002	0.2	0.1	0.01	0.8	0.01	0.1	0.03	5.4	0.01	0.01	**9.2**
	max	0.1	0.02	7.4	4.1	1.2	5.2	0.02	0.2	0.1	0.01	9.0	0.1	0.7	0.03	6.4	0.01	0.1	**35**
**PB Mince (n=6)**																			
	mean	0.1	0.02	0.7	0.2	3.3	0.9	0.01	0.3	0.1	0.01	17	0.3	0.5	0.1	1.0	0.03	0.1	**25**
	median	0.1	0.02	0.7	0.2	1.2	0.5	0.01	0.3	0.1	0.01	4.4	0.1	0.4	0.0	0.8	0.03	0.05	**8.7**
	SD	0.0	0.0	0.0	0.0	5.2	0.8	0.01	0.2	0.04	0.001	33	0.4	0.5	0.3	1.1	0.02	0.1	**33**
	min	0.1	0.02	0.7	0.2	1.2	0.1	0.002	0.2	0.03	0.01	0.4	0.01	0.1	0.03	0.1	0.01	0.02	**3.1**
	max	0.1	0.02	0.7	0.2	13.9	2.2	0.02	0.6	0.1	0.01	84	1.2	1.3	0.7	2.8	0.1	0.3	**108**
**Processed soy (n=3)**																			
	mean	0.5	0.02	3.5	0.4	1.2	0.6	0.1	1.3	0.4	0.01	69	0.1	0.7	0.03	0.1	0.03	0.04	**78**
	median	0.1	0.02	0.7	0.2	1.2	0.5	0.02	1.3	0.1	0.01	0.4	0.1	0.2	0.03	0.04	0.04	0.1	**4.9**
	SD	0.6	0.0	4.9	0.3	0.0	0.5	0.1	1.2	0.5	0.0	118	0.05	0.8	0.002	0.1	0.02	0.02	**119**
	min	0.1	0.02	0.7	0.2	1.2	0.1	0.002	0.2	0.03	0.01	0.4	0.01	0.2	0.03	0.04	0.01	0.01	**3.2**
	max	1.2	0.02	9.1	0.6	1.2	1.1	0.2	2.5	1.0	0.01	205	0.1	1.6	0.03	0.3	0.04	0.1	**225**

**PB Sausage (n=5)**																			
	mean	0.6	0.3	4.8	5.4	1.2	4.5	0.01	0.9	1.0	0.01	27	1.3	0.5	0.3	12	0.03	0.1	**60**
	median	0.1	0.02	0.7	0.2	1.2	0.5	0.01	0.8	0.1	0.01	25	0.8	0.3	0.2	2.3	0.01	0.03	**32**
	SD	0.8	0.7	6.2	10.1	0.0	9.0	0.01	0.6	2.0	0.0	24	1.4	0.4	0.4	20	0.04	0.1	**35**
	min	0.1	0.02	0.7	0.2	1.2	0.5	0.002	0.2	0.1	0.01	3.7	0.3	0.2	0.03	0.6	0.01	0.02	**7.8**
	max	1.8	1.5	15	23	1.2	21	0.02	1.7	4.5	0.01	65	3.6	1.0	1.1	47	0.1	0.2	**187**
**PB Various (n=5)**																			
	mean	0.1	0.02	0.7	0.2	1.2	0.5	0.002	1.9	0.1	0.1	34	0.9	0.8	2.2	2.9	1.6	0.1	**47**
	median	0.1	0.02	0.7	0.2	1.2	0.5	0.002	1.2	0.1	0.01	18	1.0	0.4	0.1	2.3	0.02	0.1	**26**
	SD	0.0	0.0	0.0	0.0	0.0	0.0	0.0	2.3	0.0	0.1	36	0.6	0.9	4.8	3.5	3.4	0.1	**37**
	min	0.1	0.02	0.7	0.2	1.2	0.5	0.002	0.2	0.1	0.01	3.3	0.3	0.1	0.03	0.1	0.01	0.01	**6.7**
	max	0.1	0.02	0.7	0.2	1.2	0.5	0.002	6.0	0.1	0.3	89	1.9	2.4	10.7	8.7	7.7	0.2	**130**
**PB Cold meat (n=8)**																			
	mean	0.7	0.1	0.8	0.3	36	0.6	0.03	1.1	6.9	0.1	22	0.8	0.7	0.1	0.7	0.1	0.2	**71**
	median	0.1	0.02	0.7	0.2	1.2	0.5	0.01	0.6	0.1	0.01	18	0.8	0.5	0.03	0.6	0.02	0.1	**24**
	SD	1.4	0.1	0.3	0.1	89	0.3	0.04	1.9	19	0.2	22	0.5	0.5	0.2	0.6	0.1	0.3	**93**
	min	0.1	0.02	0.7	0.2	1.2	0.2	0.002	0.01	0.1	0.01	0.1	0.03	0.2	0.01	0.01	0.01	0.01	**1.5**
	max	4.2	0.2	1.2	0.5	254	1.2	0.1	5.6	53	0.5	63	1.7	1.5	0.6	1.5	0.2	0.8	**389**
	**Total mean**	0.3	0.1	1.9	1.2	24	1.9	0.02	0.8	1.3	0.03	22	0.6	0.5	0.4	3.2	0.2	0.1	**60**

Table S 6. Individual PFR concentrations in ng/g ww in NPBF samples.

Sample-ID	TEP	TCEP	TCIPP	TiBP	TDCIPP	TnBP	V6	TPhP	TBOEP	TDBPP	EHDPHP	TpTP	iDPP	RDP	TEHP	TBuPhP	BDP	∑PFRs
PBP-01	<1	<0.5	4.2	<1	<10	<0.5	<0.1	<0.3	0.8	<0.01	0.8	<0.15	<0.3	<0.15	<0.15	<0.15	<0.1	**5.8**
PBP-02	<1	<0.5	<5	2.5	<10	1.8	<0.1	<0.3	<0.3	<0.01	<0.5	<0.15	<0.3	<0.15	<0.15	<0.15	<0.1	**4.3**
PBP-03	<0.5	<0.5	<5	<1	<10	<2	<0.01	0.4	<2	<0.01	4.1	0.7	1.6	<0.8	0.4	<0.02	0.1	**7.3**
PBP-04	<0.5	<0.5	<5	<1	<10	<2	<0.01	0.5	<2	<0.01	90	0.9	1.0	<0.8	0.8	<0.02	<0.02	**94**
PBP-05	0.8	<0.5	<5	9.0	<10	5.9	0.01	<0.3	<2	<0.01	1.4	0.0	<0.1	<0.8	4.2	<0.02	<0.02	**21**
PBP-06	<1	<0.5	<20	<5	<20	<5	0.03	<0.8	<1	<0.01	<2	<0.15	<0.1	<0.8	<0.05	<0.02	<0.02	**0.04**
PBP-07	<0.5	<0.5	<5	<1	<10	<2	<0.01	0.6	<2	<0.01	4.8	0.1	<0.1	0.6	0.3	<0.02	<0.02	**6.4**
PBP-08	<0.5	<0.5	<5	<1	24	<2	<0.01	<0.3	<2	<0.01	<1	<0.02	<0.1	<0.8	10	<0.02	<0.02	**35**
PBP-09	<1	<0.5	10	2.9	<10	3.6	<0.1	<0.3	1.2	<0.01	2.4	<0.15	<0.3	<0.15	<0.15	<0.15	<0.1	**20**
PBP-10	<0.5	<0.5	<5	3.3	587	3.8	<0.01	<0.3	<2	<0.01	<1	0.0	0.2	<0.8	8.8	<0.02	<0.1	**603**
PBP-11	<1	<0.5	18	2.1	<10	3.6	<0.1	<0.3	<0.3	<0.01	<0.5	<0.15	<0.3	<0.15	<0.15	<0.15	<0.1	**23**
PBP-12	<0.5	<0.5	<5	<1	<10	5.3	<0.01	<0.3	<2	<0.01	<1	0.7	<0.1	<0.8	5.2	<0.02	<0.02	**11**
PBP-13	<0.5	<0.5	<5	<1	<10	<2	<0.01	<0.3	<2	<0.01	2.1	2.7	<0.1	<0.8	26.4	<0.02	0.1	**31**
PBP-14	<1	<0.5	<5	1.4	<10	<0.5	<0.1	0.8	3.1	<0.01	20	0.3	<0.3	0.3	1.1	<0.15	<0.1	**27**
PBP-15	<0.5	<0.5	<5	<1	<10	<2	<0.01	<0.3	<2	<0.01	17	0.1	<0.1	0.0	0.3	<0.02	<0.02	**18**
PBP-16	<0.5	<0.5	<5	<1	267	<2	<0.01	3.2	<2	<0.01	16	2.2	1.9	0.4	1.7	0.1	0.2	**293**
PBP-17	<1	<0.5	<5	1.2	<10	1.4	<0.1	0.3	<0.3	<0.01	0.7	<0.15	<0.3	<0.15	0.2	<0.15	<0.1	**3.9**
PBP-18	<0.5	<0.5	<5	<1	<10	<2	0.1	0.9	<2	<0.01	60	1.4	0.4	0.1	0.9	<0.02	<0.02	**63**
PBP-19	<1	<0.5	<5	<0.5	<10	<0.5	<0.1	<0.3	<0.3	<0.01	<0.5	<0.15	<0.3	<0.15	<0.15	<0.15	<0.1	**0.0**
PBP-20	1.2	<0.5	<5	<1	<10	26	<0.01	1.6	<2	<0.01	54.1	1.3	1.1	1.7	1.7	0.6	1.0	**90**
PBP-21	<0.5	<0.5	<5	<1	<10	<2	<0.01	0.8	<2	<0.01	13	0.4	0.3	0.4	0.8	<0.02	0.1	**16**
PBP-22	<0.5	<0.5	<5	<1	<10	<2	<0.01	<0.3	<2	<0.01	4.8	0.0	<0.1	<0.8	1.2	<0.02	<0.02	**6.1**
PBP-23	<0.5	4.8	<5	<1	<10	<2	<0.01	1.0	<2	<0.01	22	0.9	1.2	0.2	2.1	0.1	0.3	**33**
PBP-24	<0.5	<0.5	7.4	<1	<10	<2	0.02	<0.3	<2	<0.01	9.0	0.1	0.7	<0.8	5.4	<0.02	0.1	**23**
PBP-25	<0.5	<0.5	<5	4.1	<10	5.2	<0.01	<0.3	<2	<0.01	<1	0.0	<0.1	<0.8	6.4	<0.02	<0.02	**16**
PBP-26	<1	<0.5	<5	<0.5	14	1.4	<0.1	0.5	<0.3	<0.01	2.7	<0.15	0.7	<0.15	<0.15	<0.15	<0.1	**19**
PBP-27	<1	<0.5	<5	<0.5	<10	<0.5	<0.1	<0.3	<0.3	<0.01	<0.5	<0.15	<0.3	<0.15	<0.15	<0.15	<0.1	**0.0**
PBP-28	<0.5	<0.5	<5	<1	<10	<2	<0.01	0.6	<2	<0.01	84	1.2	0.5	0.7	1.3	0.1	0.3	**89**
PBP-29	<0.5	<0.5	<5	<1	<10	<2	<0.01	0.4	<2	<0.01	6.1	0.2	0.1	<0.8	1.3	<0.02	0.03	**8.1**

PBP-30	<0.5	<0.5	<5	<1	<10	<2	<0.01	<0.3	<2	<0.01	9.4	0.1	1.3	<0.8	0.2	0.03	<0.1	**11**
PBP-31	<0.5	<0.5	<5	<1	<10	2.2	0.01	<0.3	<2	<0.01	1.5	<0.02	<0.1	<0.8	2.8	<0.02	0.03	**6.6**
PBP-32	<1	<0.5	9.1	<0.5	<10	<0.5	0.2	1.3	1.0	<0.01	<0.5	<0.15	<0.3	<0.15	<0.15	<0.15	<0.1	**12**
PBP-33	<1	<0.5	<5	0.6	<10	1.1	<0.1	<0.3	<0.3	<0.01	<0.5	<0.15	<0.3	<0.15	0.3	<0.15	<0.1	**2.0**
PBP-34	1.2	<0.5	<5	<1	<10	<2	<0.01	2.5	<2	<0.01	205	<0.02	1.6	<0.8	<0.05	<0.02	<0.02	**211**
PBP-35	<0.5	<0.5	<5	<1	<10	<2	0.02	0.4	<2	<0.01	3.7	3.6	0.3	0.1	47	<0.02	0.0	**55**
PBP-36	1.8	1.5	15	23	<10	21	<0.01	0.8	4.5	<0.01	8.2	0.3	0.3	0.2	7.0	<0.02	0.0	**83**
PBP-37	0.8	<0.5	7.4	3.2	<10	<2	0.01	1.5	<2	<0.01	36	1.2	1.0	1.1	0.6	<0.02	0.0	**53**
PBP-38	<0.5	<0.5	<5	<1	<10	<2	<0.01	1.7	<2	<0.01	65	0.8	0.8	0.3	2.3	0.1	0.2	**71**
PBP-39	<0.5	<0.5	<5	<1	<10	<2	0.01	<0.3	<2	<0.01	25	0.5	0.2	<0.8	1.5	0.1	0.1	**27**
PBP-40	<0.5	<0.5	<5	<1	<10	<2	<0.01	1.5	<2	0.3	7.7	1.0	<0.1	10.7	0.1	7.7	<0.02	**29**
PBP-41	<0.5	<0.5	<5	<1	<10	<2	<0.01	<0.3	<2	<0.01	3.3	0.3	0.2	<0.8	0.2	<0.02	<0.02	**4.0**
PBP-42	<0.5	<0.5	<5	<1	<10	<2	<0.01	0.7	<2	<0.01	18	1.2	0.4	0.2	3.1	0.1	0.2	**24**
PBP-43	<0.5	<0.5	<5	<1	<10	<2	<0.01	1.2	<2	<0.01	89	0.4	1.0	0.1	8.7	0.03	0.1	**101**
PBP-44	<0.5	<0.5	<5	<1	<10	<2	<0.01	6.0	<2	<0.01	53	1.9	2.4	<0.8	2.3	<0.02	0.2	**66**
PBP-45	<0.5	<0.5	<5	<1	33	<2	0.01	0.6	<2	<0.01	24	1.7	0.4	<0.8	1.5	0.01	0.1	**62**
PBP-46	<0.5	<0.5	<5	<1	254	<2	<0.01	5.6	<2	<0.01	42	0.8	0.2	<0.8	0.4	<0.02	0.03	**303**
PBP-47	<0.5	<0.5	<5	<1	<10	<2	<0.01	1.2	<2	<0.01	27	0.9	1.1	0.1	1.4	<0.02	0.1	**32**
PBP-48	<0.5	<0.5	<5	<1	<10	<2	0.03	0.3	<2	<0.01	63	0.8	1.5	0.6	0.8	0.2	0.8	**68**
PBP-49	4.2	<0.5	<5	<1	<10	<2	<0.01	0.7	<2	<0.01	13	1.3	0.4	<0.8	1.1	0.01	0.1	**21**
PBP-50	<0.5	<0.5	<5	<1	<10	<2	<0.01	0.5	<2	<0.01	3.4	0.5	0.3	<0.8	0.2	<0.02	<0.02	**5.0**
PBP-51	<0.5	<0.5	<5	<1	<10	<2	<0.01	1.2	<2	<0.01	53	0.5	0.5	0.3	1.1	0.01	0.1	**57**
PBP-52	<0.5	<0.5	<5	<1	<10	<2	<0.01	<0.3	<2	0.4	1.7	0.4	<0.1	<0.8	0.6	<0.01	<0.02	**2.7**

Table S 7. Descriptive statistics for LP and AP contamination in ng/g ww per NPBF category.

		DMP	DEP	DnBP	DPP	BBzP	DiBP	DEHP	DINP	DIDP	∑LPs	ATEC	DIBA	CDPHP	ATBC	DBS	DEHA	BTHC	THTM	TOTM	DEHT	DINCH	∑APs	∑plasticizers
**DF (%)**		60	77	19	54	31	19	23	25	40		63	63	88	15	33	15	40	48	12	12	19		
**PB Burger (n=7)**																								
	mean	6.2	17	29	0.5	0.9	9.0	28	20	10	**120**	5.2	0.6	1.0	5.8	0.9	24	0.1	0.003	0.4	15	2.8	**55**	**176**
	median	5.3	12	34	0.3	0.2	3.4	28	5.0	9.5	**98**	3.3	0.3	0.7	4.5	1.0	1.5	0.04	0.004	0.1	8.4	3.8	**24**	**121**
	SD	5.8	19	24	0.5	0.9	17	0.0	20	7.5	**42**	5.1	0.4	0.8	1.6	0.6	39	0.1	0.001	0.8	8.3	1.4	**40**	**58**
	min	0.6	1.5	3.8	0.1	0.2	1.7	27.6	5.0	2.0	**42**	1.9	0.2	0.1	4.5	0.2	1.5	0.004	0.002	0.1	8.4	0.2	**17**	**60**
	max	18	57	75	1.4	2.3	48	27.6	54	22	**306**	16	1.1	2.1	7.5	1.9	91	0.2	0.004	2.3	24	3.8	**150**	**456**
**PB Cheese (n=6)**																								
	mean	2.5	19	29	1.9	4.4	18	524	3.8	9.8	**612**	1.6	1.5	0.6	177	0.7	10	0.1	0.2	9.9	339	2.4	**543**	**1155**
	median	2.1	22	24	1.3	3.0	20	610	3.8	6.2	**692**	1.0	0.8	0.4	58	0.7	14	0.0	0.2	0.1	451	3.2	**529**	**1222**
	SD	1.5	8.9	19	2.3	4.1	13	266	1.4	7.5	**267**	1.9	1.5	0.6	312	0.5	6.7	0.1	0.1	20	257	1.8	**405**	**485**
	min	1.2	3.8	13	0.0	0.9	1.7	28	2.5	4.0	**55**	0.04	0.3	0.2	7.5	0.2	1.5	0.0	0.0	0.1	8.4	0.2	**18**	**73**
	max	4.5	26	58	6.0	9.9	32	718	5.0	20	**879**	4.4	3.9	1.6	805	1.6	15	0.2	0.3	51	608	3.8	**1495**	**2374**
**PB Chicken (n=10)**																								
	mean	4.6	19	25	0.5	1.2	18	65	15	11	**159**	9.2	1.9	1.9	6.3	1.6	2.9	0.1	0.1	0.6	274	2.6	**301**	**460**
	median	3.3	7.0	34	0.3	1.2	3.4	69	5.0	4.5	**128**	6.2	0.9	0.9	7.5	1.0	1.5	0.1	0.0	0.1	16	3.8	**38**	**166**
	SD	4.4	23	18	0.6	1.0	26	50	22	13	**69**	10	3.6	2.3	1.5	2.3	4.3	0.1	0.1	1.6	817	1.8	**817**	**820**
	min	0.6	1.5	3.8	0.02	0.2	3.4	28	2.5	2.0	**42**	0.1	0.2	0.1	4.5	0.2	1.5	0.004	0.002	0.1	8.4	0.2	**15**	**57**
	max	13	69	57	1.9	2.4	81	196	62	42	**524**	33	12	7.0	7.5	7.6	15	0.3	0.4	5.2	2599	4.3	**2692**	**3216**
**PB Fish (n=2)**																								
	mean	1.1	6.9	34	0.1	0.2	3.4	69	21	2.0	**138**	2.1	0.9	0.3	4.5	1.0	1.5	0.004	0.004	0.1	24	3.8	**38**	**176**
	median	1.1	6.9	34	0.1	0.2	3.4	69	21	2.0	**138**	2.1	0.9	0.3	4.5	1.0	1.5	0.004	0.004	0.1	24	3.8	**38**	**176**
	SD	0.2	0.4	0.0	0.1	0.0	0.0	0.0	23	0.0	**23**	0.8	0.0	0.0	0.0	0.0	0.0	0.0	0.0	0.0	0.0	0.0	**0.8**	**23**
	min	1.0	6.6	34	0.1	0.2	3.4	69	5.0	2.0	**121**	1.5	0.9	0.3	4.5	1.0	1.5	0.004	0.004	0.1	24	3.8	**38**	**159**
	max	1.2	7.2	34	0.2	0.2	3.4	69	37	2.0	**155**	2.6	0.9	0.3	4.5	1.0	1.5	0.004	0.004	0.1	24	3.8	**39**	**193**
**PB Mince (n=6)**																								
	mean	1.4	12	22	0.2	0.7	2.8	169	14	15	**236**	1.4	1.4	2.1	41	0.6	6.0	0.1	0.1	3.4	16	8.0	**80**	**316**
	median	1.2	6.0	24	0.1	0.5	3.4	69	5.0	5.5	**115**	1.5	0.9	0.4	6.0	0.6	1.5	0.004	0.004	0.1	16	3.8	**31**	**146**
	SD	1.0	12	14	0.2	0.8	0.9	191	15	21	**193**	0.8	1.8	3.9	86	0.5	7.0	0.1	0.1	8.0	8.5	10	**88**	**212**
	min	0.6	1.5	3.8	0.02	0.2	1.7	28	2.5	2.0	**40**	0.1	0.2	0.1	4.5	0.2	1.5	0.004	0.002	0.1	8.4	3.8	**19**	**59**
	max	3.2	30	34	0.5	2.1	3.4	466	39	56	**635**	2.5	4.9	10	217	1.0	15	0.3	0.2	20	24	29	**324**	**958**
**Processed soy (n=3)**																								
	mean	0.6	2.6	14	0.1	0.9	3.4	181	47	5.2	**255**	1.9	0.9	0.1	535	0.4	6.0	0.1	0.003	0.1	14	4.5	**562**	**817**
	median	0.6	1.5	3.8	0.1	1.3	3.4	128	61	6.8	**206**	1.9	0.9	0.1	260	0.2	1.5	0.1	0.002	0.1	8.4	3.8	**277**	**483**
	SD	0.0	1.8	18	0.0	0.7	0.0	145	37	2.8	**151**	0.0	0.0	0.0	706	0.5	7.8	0.1	0.001	0.0	9.0	2.6	**706**	**722**
	min	0.6	1.5	3.8	0.1	0.2	3.4	69	5.0	2.0	**86**	1.9	0.9	0.1	7.5	0.2	1.5	0.1	0.002	0.1	8.4	2.4	**23**	**109**
	max	0.6	4.6	34	0.1	1.4	3.4	345	76	6.9	**472**	1.9	0.9	0.1	1337	1.0	15	0.2	0.004	0.1	24	7.3	**1387**	**1860**
**PB Sausage (n=5)**																								
	mean	5.4	18	70	0.2	2.1	15	159	21	17	**308**	5.8	0.7	1.2	188	0.9	45	0.2	2.3	1.2	73	4.9	**323**	**631**
	median	2.0	18	34	0.1	0.9	3.4	69	5.0	4.0	**136**	1.9	0.7	0.5	7.5	1.0	15	0.1	0.1	0.1	24	3.8	**55**	**191**
	SD	7.3	10	90	0.3	3.6	29	225	39	21	**249**	10	0.3	1.8	407	0.5	82	0.2	3.6	2.4	118	2.4	**431**	**498**
	min	1.2	10	13	0.1	0.2	1.7	28	2.5	2.0	**59**	0.03	0.3	0.2	4.5	0.2	1.5	0.0	0.0	0.1	8.4	3.8	**19**	**78**
	max	18	36	227	0.8	8.5	67	561	90	50	**1058**	24	1.2	4.4	915	1.4	191	0.4	8.4	5.6	284	9.2	**1445**	**2503**
**PB Various (n=5)**																								
	mean	7.0	19	136	0.3	0.6	2.4	61	5.8	3.2	**235**	17	1.0	4.0	6.3	0.7	57	0.03	1.5	0.1	21	3.8	**112**	**347**
	median	1.2	15	13	0.1	0.9	1.7	69	5.0	4.0	**110**	0.1	0.9	1.6	7.5	0.9	15	0.02	0.3	0.1	24	3.8	**54**	**164**
	SD	9.2	16	169	0.3	0.4	0.9	19	4.7	1.1	**171**	28	0.7	6.0	1.6	0.4	59	0.04	2.2	0.0	7.0	0.0	**66**	**184**
	min	1.2	3.8	13	0.02	0.2	1.7	28	2.5	2.0	**52**	0.02	0.5	0.1	4.5	0.2	15	0.004	0.004	0.1	8.4	3.8	**33**	**85**
	max	22	46	353	0.6	0.9	3.4	69	14	4.0	**513**	64	2.3	15	7.5	1.0	139	0.1	5.0	0.1	24	3.8	**261**	**774**
**PB Cold meat (n=8)**																								

mean	2.7	26	19	0.1	1.5	2.1	87	6.1	5.9	**150**	0.7	1.9	1.3	141	3.7	12	0.02	2.7	0.1	14	6.3	**184**	**333**
median	1.2	16	13	0.0	0.9	1.7	28	2.5	4.0	**67**	0.1	1.4	0.9	7.5	0.8	15	0.02	2.6	0.1	8.4	3.8	**41**	**108**
SD	2.6	34	9.7	0.2	2.3	0.8	135	8.3	7.0	**140**	1.4	1.1	1.4	381	6.3	6.2	0.03	2.0	0.0	8.1	7.0	**381**	**406**
min	1.1	1.5	13	0.02	0.2	1.7	28	2.5	2.0	**50**	0.01	0.9	0.3	4.5	0.4	1.5	0.004	0.004	0.1	8.4	3.8	**20**	**70**
max	7.8	107	34	0.5	7.2	3.4	418	27	23	**627**	4.1	3.7	4.7	1083	18	15	0.1	5.0	0.1	24	24	**1181**	**1808**
**Total mean**	3.8	17	39	0.5	1.5	9.4	144	15	9.5		5.3	1.3	1.5	99	1.4	18	0.1	0.8	1.9	109	4.3		

Table S 8. Individual LP and AP concentrations in ng/g ww in NPBF samples.

Sample-ID	DMP	DEP	DnBP	DPP	BBzP	DiBP	DEHP	DINP	DIDP	∑LPs	ATBC	DBS	DEHA	BTHC	THTM	TOTM	DEHT	DINCH	ATEC	DIBA	CDPHP	∑APs	∑plasticizers
PBP-01	5.3	14	19	0.7	2.3	<20	<120	<20	12	**54**	<50	<0.5	<10	0.1	0.03	<1	<70	2.2	<3	0.3	0.3	**2.8**	**57**
PBP-02	<1	12	<20	<0.15	<0.5	<20	<120	<20	9.5	**22**	<50	<0.5	<10	<0.01	<0.01	<1	<70	<1	<3	<0.15	<0.15	**0.0**	**22**
PBP-03	8.3	57	75	<0.15	<0.5	48	<120	<20	22	**210**	<50	1.9	<10	0.0	<0.01	2.3	<70	1.7	<3	1.1	2.1	**9.1**	**219**
PBP-04	3.8	19	<180	<0.15	<0.5	<20	<120	54	<5	**77**	<30	<3	67	<0.1	<0.02	<1	<70	<20	3.7	0.3	0.7	**72**	**149**
PBP-05	1.6	4.5	<180	0.8	1.6	<20	<300	23	17	**49**	<30	<3	<10	<0.1	<0.02	<1	<200	<20	3.3	0.2	0.6	**4.1**	**53**
PBP-06	18	<2	<180	1.4	1.5	<20	<300	39	7.3	**68**	<30	<3	91	<0.1	<0.02	<1	<200	<20	7.7	0.3	1.2	**101**	**168**
PBP-07	5.7	10	<180	0.3	<0.5	<20	<300	<20	<5	**16**	<30	<3	<10	0.2	<0.02	<1	<200	<20	16	<0.15	1.9	**18**	**34**
PBP-08	3.0	21	58	2.4	8.4	31	471	<20	8.4	**603**	<50	<0.5	13	0.01	0.01	<1	<70	<1	<3	0.3	0.6	**14**	**616**
PBP-09	3.8	26	40	6.0	9.9	32	517	<20	18	**653**	<50	<0.5	<10	0.1	0.1	51	608	2.5	4.4	0.6	1.6	**668**	**1321**
PBP-10	<2	<5	<70	0.0	<3	17	704	<10	<10	**721**	805	<0.5	<100	0.2	0.3	<1	490	<20	0.0	<0.5	0.2	**1296**	**2017**
PBP-11	4.5	23	<20	2.6	5.1	<20	<120	<20	20	**55**	<50	0.7	<10	0.1	0.1	7.7	476	<1	3.2	0.4	1.1	**489**	**545**
PBP-12	<2	12	<70	0.1	<3	23	708	<10	<10	**743**	128	0.7	<100	<0.05	0.2	<1	<200	<20	0.0	3.9	0.2	**133**	**876**
PBP-13	<2	25	<70	0.2	<3	<10	718	<10	<10	**743**	109	1.6	<100	<0.05	0.3	<1	426	<20	0.1	2.7	0.2	**540**	**1283**
PBP-14	8.4	40	36	1.4	2.4	32	<120	50	25	**195**	<50	7.6	<10	0.2	0.1	5.2	<70	2.4	7.7	0.8	2.2	**26**	**221**
PBP-15	3.1	<2	<180	0.7	1.8	<20	<300	22.5	<5	**28**	<30	<3	<10	0.1	<0.02	<1	<200	<20	12	<0.15	1.1	**14**	**19**
PBP-16	13	69	57	1.9	1.5	39	196	62	42	**482**	<50	3.1	<10	0.1	0.1	<1	<70	4.3	14	1.1	7.0	**29**	**511**
PBP-17	1.2	7.5	<20	<0.15	<0.5	<20	<120	<20	11	**20**	<50	<0.5	<10	0.1	<0.01	<1	<70	<1	<3	<0.15	0.2	**0.2**	**20**
PBP-18	9.9	<2	<180	0.8	2.4	<20	<300	<20	13	**26**	<30	<3	<10	0.2	0.1	<1	<200	<20	33	0.6	1.5	**36**	**62**
PBP-19	<1	<2	<20	<0.15	<0.5	<20	<120	<20	<5	**0.0**	<50	<0.5	<10	0.05	<0.01	<1	<70	<1	<3	<0.15	<0.15	**0.05**	**0.05**
PBP-20	3.5	20	<20	<0.15	1.9	<20	<120	<20	<5	**25**	<50	<0.5	<10	0.3	0.1	<1	<70	<1	<3	0.3	0.5	**1.3**	**26**
PBP-21	3.5	6.5	<180	<0.15	<0.5	<20	<300	<20	5.1	**15**	<30	<3	<10	<0.1	<0.02	<1	<200	<20	15	0.2	0.5	**16**	**31**
PBP-22	1.3	<2	<180	0.4	<0.5	<20	<300	<20	<5	**1.7**	<30	<3	<10	<0.1	<0.02	<1	<200	<20	4.8	<0.15	0.8	**5.6**	**7.3**
PBP-23	<2	38	<70	<0.03	<3	81	<300	<10	<10	**119**	<50	1.1	<100	<0.05	0.4	<1	2599	<20	0.1	12.0	4.8	**2618**	**2737**
PBP-24	1.0	6.6	<180	<0.15	<0.5	<20	<300	37.3	<5	**45**	<30	<3	<10	<0.1	<0.02	<1	<200	<20	1.5	<0.15	0.3	**1.8**	**47**
PBP-25	1.2	7.2	<180	0.2	<0.5	<20	<300	<20	<5	**8.6**	<30	<3	<10	<0.1	<0.02	<1	<200	<20	2.6	<0.15	0.3	**2.9**	**12**
PBP-26	<2	<5	<70	0.1	<3	<10	352	27	<10	**379**	217	<0.5	<100	0.3	0.1	<1	<70	<20	0.1	0.9	0.4	**219**	**598**
PBP-27	<1	<2	<20	<0.15	<0.5	<20	<120	39	7.0	**46**	<50	<0.5	<10	<0.01	<0.01	20	<70	29	<3	0.5	0.4	**49**	**95**
PBP-28	3.2	22	<70	<0.03	<3	<10	<120	<10	<10	**25**	<50	<0.5	<100	0.2	0.2	<1	<70	<20	1.1	4.9	10.1	**17**	**42**
PBP-29	1.2	6.4	<180	0.5	2.1	<20	466	<20	56	**533**	<30	<3	<10	<0.1	<0.02	<1	<200	<20	2.5	<0.15	<0.15	**2.5**	**535**
PBP-30	1.6	30	<180	0.2	<0.5	<20	<300	<20	<5	**32**	<30	<3	<10	<0.1	<0.02	<1	<200	<20	1.9	0.2	1.4	**3.4**	**35**
PBP-31	<1	5.6	<180	0.3	<0.5	<20	<300	<20	16	**22**	<30	<3	<10	<0.1	<0.02	<1	<200	<20	1.1	<0.15	0.3	**1.4**	**24**
PBP-32	<1	4.6	<20	<0.15	1.3	<20	345	61	6.9	**419**	260	<0.5	15	0.1	<0.01	<1	<70	2.4	<3	<0.15	<0.15	**277**	**696**
PBP-33	<1	<2	<20	<0.15	<0.5	<20	128	76	6.8	**210**	<50	<0.5	<10	0.1	0.0	<1	<70	7.3	<3	<0.15	<0.15	**7.4**	**218**
PBP-34	<1	<2	<180	<0.15	1.4	<20	<300	<20	<5	**1.4**	1337	<3	<10	0.2	<0.02	<1	<200	<20	<3	<0.15	<0.15	**1337**	**1338**
PBP-35	4.1	18	63	<0.15	8.5	67	561	90	50	**860**	<50	1.4	16	0.3	0.1	5.6	284	9.2	<3	0.5	0.3	**317**	**1178**
PBP-36	2.0	10	<180	<0.15	<0.5	<20	<300	<20	23.4	**35**	<30	<3	<10	<0.1	<0.02	<1	<200	<20	2.7	0.3	0.8	**3.8**	**39**
PBP-37	<2	11	<70	0.1	<3	<10	<120	<10	<10	**11**	915	0.7	<100	<0.05	3.0	<1	<70	<20	0.2	1.2	0.5	**921**	**932**

PBP-38	18	36	227	0.8	<0.5	<20	<300	<20	<5	**282**	<30	<3	<10	0.4	0.1	<1	<200	<20	24	0.9	4.4	**30**	**312**
PBP-39	<2	18	<70	0.1	<3	<10	<300	<10	<10	**18**	<50	<0.5	191	0.1	8.4	<1	<200	<20	0.0	0.7	0.2	**200**	**218**
PBP-40	<2	15	<70	<0.03	<3	<10	<300	<10	<10	**15**	<50	0.7	<100	0.1	5.0	<1	<200	<20	0.1	<0.5	1.6	**7.4**	**22**
PBP-41	<2	0.0	<70	<0.03	<3	<10	<120	13.9	<10	**14**	<50	<0.5	<100	<0.05	0.3	<1	<70	<20	0.0	2.3	0.1	**2.7**	**17**
PBP-42	<2	9.2	<70	0.1	<3	<10	<300	<10	<10	**9.3**	<50	0.9	<100	<0.05	2.4	<1	<200	<20	0.0	0.6	0.4	**4.2**	**14**
PBP-43	9.2	22	285	0.5	<0.5	<20	<300	<20	<5	**317**	<30	<3	139	<0.1	<0.02	<1	<200	<20	21	0.5	3.2	**164**	**481**
PBP-44	22	46	353	0.6	<0.5	<20	<300	<20	<5	**422**	<30	<3	100	<0.1	<0.02	<1	<200	<20	64	<0.15	15	**178**	**600**
PBP-45	<2	13	<70	<0.03	<3	<10	<120	26.5	<10	**39**	1083	18	<100	0.1	2.2	<1	<70	<20	0.1	1.8	0.5	**1106**	**1145**
PBP-46	<2	15	<70	<0.03	7.2	<10	<120	<10	23.1	**46**	<50	0.4	<100	<0.05	4.5	<1	<70	23.6	0.0	1.0	1.3	**31**	**76**
PBP-47	7.8	33	<70	<0.03	<3	<10	<120	<10	<10	**40**	<50	0.4	<100	<0.05	5.0	<1	<70	<20	0.1	3.7	0.8	**10**	**50**
PBP-48	5.5	17	<70	<0.03	<3	<10	<120	<10	<10	**22**	<50	0.6	<100	<0.05	2.6	<1	<70	<20	0.0	2.7	1.8	**7.7**	**30**
PBP-49	<2	17	<70	<0.03	<3	<10	<120	<10	<10	**17**	<50	8.0	<100	<0.05	4.6	<1	<70	<20	0.1	3.2	0.5	**16**	**34**
PBP-50	1.1	4.7	<180	0.5	<0.5	<20	<300	<20	<5	**6.3**	<30	<3	<10	<0.1	<0.02	<1	<200	<20	1.0	<0.15	4.7	**5.7**	**12**
PBP-51	<2	107	<70	<0.03	<3	<10	418	<10	<10	**524**	<50	0.4	<100	<0.05	2.7	<1	<200	<20	<0.01	<0.5	0.3	**3.4**	**528**
PBP-52	2.0	<2	<180	0.4	<0.5	<20	<300	<20	<5	**2.4**	<30	<3	<10	<0.1	<0.02	<1	<200	<20	4.1	<0.15	0.9	**5.0**	**7.4**

Table S 9. Correlation coefficients and p-values for correlations between individual and total PFR, LP and AP concentrations in NPBFs and fat content of the samples.

compound	correlation coefficient	*p*-value	compound	correlation coefficient	*p*-value
**PFRs**			**plasticizers**		
TEP	-0.2	0.6	**LPs**		
TCEP	0.1	0.4	DMP	0.3	0.06
TCIPP	0.1	0.3	DEP	0.4	**0.03***
TiBP	-0.1	0.5	DnBP	0.4	**0.01****
TDCIPP	0.2	0.5	DPP	0.3	**0.01****
TnBP	0.1	0.9	BBzP	0.2	**0.04***
V6	-0.1	0.7	DEHP/DEHT	0.5	**<0.001****
TPhP	0.0	0.7	DINP	-0.2	0.3
TBOEP	0.3	0.6	DIDP	0.0	1.0
TDBPP	-0.1	0.7	DiBP	0.2	**0.02***
EHDPHP	0.1	0.8	**APs**		
TpTP	0.2	0.1	DBS	0.3	0.2
iDPP	0.1	0.6	DEHA	0.3	**0.04***
RDP	0.0	0.3	BTHC	0.1	0.5
TEHP	0.3	**0.02***	THTM	0.2	1.0
TBuPhP	0.0	0.5	TOTM	0.1	0.5
BDP	0.2	0.7	ATEC	0.0	0.6
**∑PFRs**	0.3	0.1	DIBA	-0.2	0.8
			CDPHP	0.3	0.1
			DINCH	-0.1	0.4
			ATBC	0.0	0.4
			**∑plasticizers**	0.5	**<0.001****

Table S 10. Descriptive statistics in ng/g plastic and detection frequencies of PFRs in FCM samples.

		TEP	TCEP	TCIPP	TiBP	TDCIPP	TnBP	V6	TPhP	TBOEP	TDBPP	EHDPHP	TpTP	iDPP	RDP	TEHP	TBuPhP	BDP	∑PFRs
**DF (%)**		33	56	14	6	28	17	47	61	69	44	14	17	61	83	83	78	31	
**PE (n=4)**																			
	mean	1.7	15	90	1	31	12	3.4	57	23	2.2	71	2.6	11	16	19	3.1	21	**381**
	median	1.6	17	120	0.6	42	1.2	5	85	31	3.3	64	0.5	5.3	16	16	3.5	27	**438**
	SD	0.2	3.6	51	0.7	19	20	2.8	48	14	1.9	13	3.7	10	1.3	5.5	0.7	8.9	**204**
	min	1.6	11	32	0.6	8.5	1.2	0.2	1.8	6.2	0.004	64	0.5	5.3	14	16	2.2	11	**176**
	max	2	17	120	1.9	42	35	5	85	31	3.3	86	6.9	23	16	26	3.5	27	**529**
**PET (n=37)**																			
	mean	3.6	4.4	4.9	0.6	8.3	2.3	2.7	14	4.5	1.1	3	0.9	1.1	3.8	2.9	2.2	4.1	**65**
	median	1.6	0.8	4.2	0.6	5.6	1.2	0.2	2.7	0.4	0.004	0.7	0.5	0.1	0.6	1.3	1.5	1.6	**24**
	SD	4.6	6.9	4.5	0.2	7.9	5.6	4.7	23	5.6	1.9	14	1.3	4	5.1	4.7	2.1	4.1	**101**
	min	1.6	0.8	4.2	0.6	5.6	1.2	0.2	1.8	0.4	0.004	0.7	0.5	0.1	0.1	0.1	0.4	1.6	**20**
	max	18	24	32	1.9	39	35	17	71	19	7.6	86	6.9	23	15	26	10	11	**442**
**PP (n=9)**																			
	mean	5.8	17	30	1.4	13	4.8	6.4	11	6.4	3.4	14	1.5	1.8	2.1	6.2	4.5	5.8	**134**
	median	1.6	9.1	4.2	0.6	5.6	1.2	7.9	6.5	5	3	0.7	1.3	1.6	2.7	6.4	5.1	1.6	**64**
	SD	7.1	19	77	2.3	12	11	4	17	5.3	2.8	39	1	2.6	1.6	3.5	3.3	5.1	**213**
	min	1.6	0.8	4.2	0.6	5.6	1.2	0.2	1.8	2.9	0.004	0.7	0.5	0.1	0.1	0.4	0.1	1.3	**22**
	max	18	64	235	7.4	34	33	11	54	20	6.1	117	2.6	8.6	3.6	9.2	7.4	11	**643**
	**Total mean**	4.1	7.4	18	0.8	19	9.3	3.5	17	6.1	1.5	8.6	2.4	1.6	3.9	5.7	2.8	5.4	**116**

Table S 11. Individual PFR concentrations in ng/g plastic in FCM samples.

Sample-ID	TEP	TCEP	TCIPP	TiBP	TDCIPP	TnBP	V6	TPhP	TBOEP	TDBPP	EHDPHP	TpTP	iDPP	RDP	TEHP	TBuPhP	BDP
FCM-01	<5.0	9.1	<30	<10	<20	<7	7.9	6.5	5.0	6.1	<5	2.6	1.6	3.6	9.2	7.4	11
FCM-02	6.0	12	<30	<10	<20	<7	9.7	13	6.2	5.8	<5	3.3	1.8	15	8.6	6.1	10
FCM-03	<5.0	<1.5	<30	<10	<20	<7	<0.5	16	15	<0.01	<5	<3	<0.1	7.6	1.0	0.9	<5
FCM-04	<5.0	<1.5	<30	<10	<20	<7	0.8	71	<0.5	0.6	<5	<3	<0.1	10.8	1.6	2.1	11
FCM-05	<5.0	<1.5	<30	<10	<20	<7	<0.5	<3	<0.5	0.3	<5	<3	<0.1	<0.1	1.2	1.5	<5
FCM-06	<5.0	<1.5	<30	<10	<20	<7	<0.5	<3	<0.5	<0.01	<5	<3	0.2	0.2	0.7	0.9	<5
FCM-07	<5.0	5.7	107	<10	97	119	<0.5	45	21	<0.01	51	77	11	5.2	84	4.2	<5
FCM-08	15	24	<30	<10	28	7.5	17	7.7	8.6	7.6	<5	3.2	1.3	2.8	12	10	10
FCM-09	10	18	<30	<10	24	<7	12	4.4	5.2	3.9	<5	<3	0.9	1.2	6.8	4.8	<5
FCM-10	<5.0	<1.5	<30	<10	<20	<7	<0.5	<3	14	<0.01	<5	<3	<0.1	0.2	<0.5	1.3	<5
FCM-11	15	17	<30	<10	<20	<7	12.5	2.7	5.1	2.4	<5	<3	0.6	0.6	5.3	4.8	<5
FCM-12	<5.0	<1.5	<30	<10	<20	<7	<0.5	<3	11	<0.01	<5	<3	<0.1	0.1	0.8	1.2	<5
FCM-13	<5.0	<1.5	<30	<10	<20	<7	<0.5	<3	11	<0.01	<5	<3	<0.1	0.1	0.6	0.8	<5
FCM-14	<5.0	9.1	<30	<10	<20	<7	7.9	6.5	5.0	6.1	<5	2.6	1.6	3.6	9.2	7.4	11
FCM-15	6.0	12	<30	<10	<20	<7	9.7	13	6.2	5.8	<5	3.3	1.8	15	8.6	6.1	10
FCM-16	18	21	<30	<10	<20	<7	11	2.6	2.9	2.4	<5	<3	0.2	0.6	5.4	5.1	<5
FCM-17	18	23	<30	<10	29	<7	9.8	5.0	3.4	3.0	<5	<3	0.2	0.4	6.2	4.9	<5
FCM-18	<5.0	<1.5	<30	<10	<20	<7	<0.5	16	15	<0.01	<5	<3	0.0	7.6	1.0	0.9	<5
FCM-19	15	17	<30	<10	<20	<7	12.5	2.7	5.1	2.4	<5	<3	0.6	0.6	5.3	4.8	<5
FCM-20	18	23	<30	<10	29	<7	9.8	5.0	3.4	3.0	<5	<3	0.2	0.4	6.2	4.9	<5
FCM-21	<5.0	1.9	<30	<10	<20	<7	4.6	8.3	19	4.1	<5	<3	7.8	3.4	1.6	1.0	4.6
FCM-22	<5.0	<1.5	<30	<10	<20	<7	<0.5	<3	<0.5	<0.01	<5	<3	<0.1	<0.1	0.4	<0.5	<5
FCM-23	<5.0	17	120	<10	42	<7	5.0	85	31	3.3	64	<3	5.3	17	16	3.5	27
FCM-24	<5.0	<1.5	<30	<10	<20	<7	0.8	71	<0.5	0.6	<5	<3	<0.1	11	1.6	2.1	11
FCM-25	<5.0	<1.5	<30	<10	<20	<7	<0.5	<3	<0.5	0.3	<5	<3	<0.1	<0.1	1.2	1.5	<5
FCM-26	<5.0	9.1	<30	<10	<20	<7	7.9	6.5	5.0	6.1	<5	2.6	1.6	3.6	9.2	7.4	11
FCM-27	<5.0	<1.5	<30	<10	<20	<7	<0.5	16	15	<0.01	<5	<3	<0.1	7.6	1.0	0.9	<5
FCM-28	<5.0	<1.5	<30	<10	<20	<7	0.8	71	<0.5	0.6	<5	<3	<0.1	11	1.6	2.1	11
FCM-29	<5.0	<1.5	<30	<10	<20	<7	<0.5	<3	<0.5	0.3	<5	<3	<0.1	<0.1	1.2	1.5	<5

FCM-30	<5.0	<1.5	<30	<10	23	<7	<0.5	5.7	20	<0.01	<5	<3	1.0	<0.1	0.7	0.4	<5
FCM-31	<5.0	<1.5	<30	<10	<20	<7	<0.5	<3	<0.5	<0.01	<5	<3	<0.1	<0.1	<0.5	0.4	<5
FCM-32	13	27	116	<10	334	<7	9.2	30	8.8	<0.01	34	<3	5.1	2.8	12	8.7	19
FCM-33	6.3	6.3	<30	<10	<20	8.8	2.3	17	1.6	1.0	<5	<3	0.2	0.9	3.3	3.4	<5
FCM-34	6.4	64	235	7.4	34	34	<0.5	54	8.7	<0.01	117	1.3	8.6	2.7	6.4	<0.5	1.3
FCM-35	<5.0	9.1	<30	<10	<20	<7	7.9	6.5	5.0	6.1	<5	2.6	1.6	3.6	9.2	7.4	11
FCM-36	<5.0	3.4	<30	<10	<20	<7	4.5	<3	2.9	0.4	<5	<3	<0.1	0.8	<0.5	<0.5	<5
FCM-37	<5.0	<1.5	<30	<10	<20	<7	0.8	71	<0.5	0.6	<5	<3	<0.1	11	1.6	2.1	11
FCM-38	<5.0	3.1	<30	<10	<20	<7	2.1	24	0.6	1.1	<5	<3	<0.1	2.3	1.2	<0.5	<5
FCM-39	<5.0	3.5	<30	<10	<20	225	2.1	9.7	2.1	<0.01	<5	<3	1.5	0.6	1.7	<0.5	<5
FCM-40	<5.0	<1.5	<30	<10	<20	<7	<0.5	3.3	<0.5	<0.01	<5	<3	0.3	4.1	<0.5	<0.5	<5
FCM-41	<5.0	<1.5	<30	<10	<20	<7	0.8	71	<0.5	0.6	<5	<3	<0.1	11	1.6	2.1	11
FCM-42	<5.0	<1.5	<30	<10	<20	<7	<0.5	<3	11	<0.01	<5	<3	<0.1	0.1	0.8	1.2	<5
FCM-43	<5.0	<1.5	<30	<10	<20	<7	<0.5	<3	<0.5	<0.01	<5	<3	<0.1	0.1	<0.5	<0.5	<5
FCM-44	<5.0	17	120	<10	42	<7	5.0	85	31	3.3	64	<3	5.3	17	16	3.5	27
FCM-45	<5.0	<1.5	<30	<10	<20	<7	<0.5	<3	<0.5	<0.01	<5	<3	<0.1	<0.1	1.8	2.6	9.3
FCM-46	<5.0	<1.5	<30	<10	39	<7	<0.5	<3	<0.5	<0.01	<5	<3	0.3	0.1	1.6	2.3	<5
FCM-47	<5.0	<1.5	<30	<10	<20	<7	<0.5	<3	<0.5	<0.01	<5	<3	<0.1	0.2	1.3	2.1	<5
FCM-48	<5.0	1.9	<30	<10	<20	<7	<0.5	5.5	<0.5	<0.01	<5	<3	<0.1	0.4	1.4	2.4	<5
FCM-49	<5.0	<1.5	<30	<10	<20	<7	<0.5	6.7	1.6	<0.01	<5	<3	<0.1	0.7	1.4	1.9	<5
FCM-50	<5.0	<1.5	<30	<10	<20	<7	<0.5	<3	<0.5	<0.01	<5	<3	<0.1	0.2	<0.5	<0.5	<5
FCM-51	2.0	11	32	1.9	8.5	35	<0.5	<3	6.2	<0.01	86	6.9	23	14	26	2.2	11
FCM-52	<5.0	<1.5	<30	<10	<20	<7	<0.5	<3	<0.5	<0.01	<5	<3	<0.1	1.8	<0.5	<0.5	<5

Table S 12. Descriptive statitistics in ng/g plastic and detection frequencies of LP and AP contamination in FCM samples.

		DMP	DEP	DnBP	DPP	BBzP	DiBP	DEHP/DEHT	DINP	DIDP	∑LPs	ATEC	DIBA	CDPHP	ATBC	DBS	DEHA	BTHC	THTM	TOTM	DINCH	∑APs	∑plasticizers
**DF (%)**		8	39	8	72	17	31	28	39	14		8	8	67	39	58	75	14	11	17	97		
**PE (n=4)**																							
	mean	0.4	139	51	0.4	94	184	2404	202	117	**3191**	0.1	1.3	2.4	10895	24	9364	0.3	0.1	64	252	**20604**	**23795**
	median	0.4	112	4	0.4	6.1	169	935	275	21	**1523**	0.1	0.8	2.3	714	4.2	6261	0.3	0.1	15	297	**7296**	**8820**
	SD	0	46	82	0	152	26	2544	127	166	**3143**	0.1	0.9	0.2	17634	34	5375	0.02	0.1	85	79	**23208**	**26351**
	min	0.4	112	4	0.4	6.1	169	935	55	21	**1303**	0.1	0.8	2.3	714	4.2	6261	0.2	0.01	15	160	**7159**	**8462**
	max	0.4	192	146	0.4	269	213	5341	275	309	**6746**	0.3	2.4	2.6	31257	62	15571	0.3	0.1	162	297	**47356**	**54102**
**PET (n=37)**																							
	mean	1.5	50.2	7.8	0.3	2437	563	373	40	29	**3502**	0.1	0.9	0.4	1243	4	679	0.3	0.1	10	285	**2223**	**5725**
	median	0.4	5.9	4	0.2	0.5	3.1	98	20	21	**153**	0.1	0.8	0.2	98	1.1	151	0.3	0.1	1.7	47	**300**	**452**
	SD	3.7	93.5	23	0.5	10302	2334	927	43	47	**13774**	0.4	0.3	0.5	5240	12	2530	0.1	0.03	29	982	**8794**	**22569**
	min	0.4	5.9	4	0.1	0.5	3.1	98	20	21	**152**	0	0.8	0.1	83	0.4	30	0.1	0.01	1.7	1.9	**118**	**271**
	max	16	269	146	2	44947	10191	5341	247	309	**61468**	2.4	2.4	2.6	31257	62	15571	0.3	0.2	162	4334	**51395**	**112864**
**PP (n=9)**																							
	mean	4.6	71	337	0.8	32	814	1434	68	76	**2837**	0.1	4	0.8	25612	4.4	340	4.1	1	8.8	90	**26065**	**28903**
	median	0.4	19	4	0.4	0.5	32	98	20	21	**195**	0.1	0.8	0.1	98	1.5	70	6.4	1	1.7	28	**207**	**402**
	SD	12	162	1000	0.7	94	2384	3594	101	110	**7459**	0.1	8.7	1.8	75858	10	840	3.7	1	15	173	**76912**	**84371**
	min	0.4	5.9	4	0.1	0.5	3.1	98	20	21	**152**	0	0.8	0.1	98	0.4	30	0.3	0.01	1.7	3.1	**134**	**286**
	max	38	503	3005	1.5	283	7170	10964	302	310	**22575**	0.3	27	5.6	227898	32	2578	9.9	2	46	549	**231148**	**253723**
	**Total mean**	1.9	59	76	0.4	1742	583	642	96	62	**9530**	0.1	1.4	0.6	7171	5.2	2300	1	0.4	9.8	384	**48892**	**58422**

Table S 13. Individual LP and AP concentrations in ng/g plastic in FCM samples.

Sample-ID	DMP	DEP	DnBP	DPP	BBzP	DEHP/ DEHT	DiBP	DINP	DIDP	ATEC	DIBA	CDPHP	ATBC	DBS	DEHA	BTHC	THTM	TOTM	DINCH
FCM-01	<5	19.4	<50	1.5	<3	<350	32	<50	<150	<1	<1	<0.01	<250	1.5	30	6.4	2.0	<10	28
FCM-02	16	<15	<50	2.0	<3	<350	<10	82	<150	<1	<1	0.9	<250	2.0	219	<2	<1	<10	4334
FCM-03	<5	<15	<50	0.2	<3	<350	<10	<50	<150	<1	<1	<0.01	<250	2.5	245	<2	<1	<10	63
FCM-04	<5	269	<50	0.2	<3	<350	<10	81	<150	<1	<1	0.2	<250	1.5	759	<2	<1	<10	133
FCM-05	<5	<15	<50	0.1	<3	<350	<10	<50	<150	<1	<1	0.2	<250	3.7	104	<2	<1	<10	53
FCM-06	<5	<15	<50	0.2	<3	<350	<10	55	<150	<1	<1	<0.01	<250	<0.5	30	<2	<1	<10	24
FCM-07	<5	106	602	0.3	89	4474	1337	1270	723	<1	<1	3.9	1680	34	1730	<2	<1	23	7129
FCM-08	<5	<15	<50	1.4	<3	<350	<10	<50	<150	2.4	<1	0.8	<250	1.6	30	<2	<1	<10	<2
FCM-09	<5	<15	<50	1.1	<3	<350	<10	<50	<150	<1	<1	0.6	<250	1.2	30	<2	<1	<10	40
FCM-10	<5	<15	<50	0.1	<3	<350	<10	<50	<150	<1	<1	0.2	<250	<0.5	179	<2	<1	<10	18
FCM-11	<5	<15	<50	0.3	<3	<350	<10	<50	<150	<1	<1	0.5	422	1.1	605	<2	<1	<10	8.4
FCM-12	<5	<15	<50	<0.1	44947	98	10191	<50	<150	<1	<1	<0.01	<250	1.1	220	<2	<1	<10	5.1
FCM-13	<5	<15	<50	<0.1	<3	<350	20	<50	<150	<1	<1	<0.01	<250	38	104	<2	<1	<10	21
FCM-14	<5	19	<50	1.5	<3	<350	32	<50	<150	<1	<1	<0.01	<250	1.5	30	6.4	2.0	<10	28
FCM-15	16	<15	<50	2.0	<3	<350	<10	82	<150	<1	<1	0.9	<250	2.0	219	<2	<1	<10	4334
FCM-16	<5	44	<50	<0.1	<10	1259	16	169	224	<0.05	3.1	<0.1	962	<0.8	131	0.8	1.0	21	59
FCM-17	<5	<15	<50	<0.1	<3	<350	<10	<50	<150	<1	<1	<0.01	<250	<0.5	70	<2	<1	<10	64
FCM-18	<5	<15	<50	0.2	<3	<350	<10	<50	<150	<1	<1	<0.01	<250	2.5	245	<2	<1	<10	64
FCM-19	<5	<15	<50	0.3	<3	<350	<10	<50	<150	<1	<1	0.5	422	1.1	605	<2	<1	<10	8.4
FCM-20	<5	<15	<50	<0.1	<3	<350	<10	<50	<150	<1	<1	0.1	<250	<0.5	70	<2	<1	<10	64
FCM-21	<5	<15	<50	<0.1	<3	890	<10	<50	<150	<1	<1	0.3	<250	<0.5	145	<2	<1	<10	59
FCM-22	<5	<15	<50	<0.1	<3	<350	<10	<50	<150	<1	<1	0.2	<250	1.1	87	<2	<1	<10	19
FCM-23	<5	113	<50	<0.1	6.1	935	167	275	<150	<1	<1	2.3	714	4.2	6261	<2	<1	15	298
FCM-24	<5	269	<50	0.2	<3	<350	<10	81	<150	<1	<1	0.2	<250	1.5	759	<2	<1	<10	133
FCM-25	<5	<15	<50	<0.1	<3	<350	<10	<50	<150	<1	<1	0.2	<250	3.7	104	<2	<1	<10	53
FCM-26	<5	19	<50	1.5	<3	<350	32	<50	<150	<1	<1	<0.01	<250	1.5	30	6.4	2.0	<10	28
FCM-27	<5	<15	<50	0.2	<3	<350	<10	<50	<150	<1	<1	<0.01	<250	2.5	245	<2	<1	<10	64

FCM-28	<5	269	<50	0.2	<3	<350	<10	81	<150	<1	<1	0.2	<250	1.5	759	<2	<1	<10	133
FCM-29	<5	<15	<50	<0.1	<3	<350	<10	<50	<150	<1	<1	0.2	<250	3.7	104	<2	<1	<10	53
FCM-30	<5	<15	<50	<0.1	<3	<350	<10	<50	<150	<1	<1	0.3	<250	<0.5	83	<2	<1	<10	3.1
FCM-31	<5	<15	<50	<0.1	<3	<350	<10	<50	<150	<1	<1	0.2	<250	1.1	87	<2	<1	<10	19
FCM-32	<5	54	<50	1.2	28	<350	239	897	687	<1	<1	2.0	85958	40	56987	2.6	6.2	<10	620
FCM-33	<5	56	<50	0.3	<3	<350	<10	104	<150	<1	<1	0.5	1621	<0.5	1344	<2	<1	<10	82
FCM-34	38	503	3004	<0.1	28	10964	7170	302	310	0.3	27	5.6	227898	32	2578	9.9	<1	47	549
FCM-35	<5	19	<50	1.5	<3	<350	32	<50	<150	<1	<1	<0.01	<250	1.5	30	6.4	2.0	<10	28
FCM-36	<5	<15	<50	0.3	<3	<350	<10	<50	<150	<1	<1	0.4	1061	<0.5	80	<2	<1	<10	25
FCM-37	<5	269	<50	0.2	<3	<350	<10	81	<150	<1	<1	0.2	<250	1.5	759	<2	<1	<10	133
FCM-38	8.2	66	<50	<0.1	0.5	663	<10	247	<150	<1	<1	0.4	<250	<0.5	151	<2	<1	<10	24
FCM-39	<5	106	<50	0.3	<3	<350	277	73	<150	<1	<1	1.1	5695	1.1	18812	<2	<0.1	<10	158
FCM-40	<5	<15	<50	<0.1	<10	2015	16	32	<150	<0.05	<2	<0.1	2096	<0.8	671	<0.5	0.2	51	42
FCM-41	<5	269	<50	0.2	<3	<350	<10	81	<150	<1	<1	0.2	<250	1.5	759	<2	<1	<10	133
FCM-42	<5	<15	<50	<0.1	44947	<350	10191	<50	<150	<1	<1	<0.01	<250	1.1	220	<2	<1	<10	5.1
FCM-43	<5	<15	<50	<0.1	<3	<350	<10	<50	<150	<1	<1	0.2	<250	1.1	87	<2	<1	<10	19
FCM-44	<5	113	<50	<0.1	6.1	935	169	275	<150	<1	<1	2.3	714	4.2	6261	<2	<1	15	298
FCM-45	<5	30	<50	<0.1	<3	<350	<10	<50	<150	<1	<1	<0.01	7938	1.2	82	<2	<1	<10	76
FCM-46	<5	39	<50	0.2	<3	<350	<10	<50	<150	<1	<1	0.3	<250	<0.5	77	<2	<1	<10	22
FCM-47	<5	<15	<50	0.2	<3	<350	<10	<50	<150	<1	<1	0.2	<250	0.7	30	<2	<1	<10	9.1
FCM-48	<5	26	<50	0.3	<3	<350	70	<50	<150	<1	<1	0.4	<250	0.8	30	<2	<1	<10	12
FCM-49	<5	<15	<50	<0.1	<10	582	<10	<50	<150	<0.05	<2	0.7	83	<0.8	75	<0.5	<0.1	<10	130
FCM-50	<5	<15	<50	<0.1	<3	<350	<10	<30	<150	<1	<1	<0.1	<250	<0.5	30	<2	<1	<10	47
FCM-51	<5	192	146	<0.1	269	5341	214	55	309	0.3	2.4	2.6	31257	62	15571	0.2	<1	162.3	160
FCM-52	<5	<15	<50	<0.1	<10	1285	<10	<30	<150	<0.05	<2	<0.1	859	<0.8	618	<0.5	<0.1	<10	38

Table S 14. Exposure and risk assessment for individual PFRs. Three separate scenarios were calculated: flexitarian (10% substitution of meat consumption with PB meat-alternatives), vegetarian (50% substitution of meat consumption with PB meat-alternatives) and vegan (100% substitution of meat, fish and cheese consumption with NPBFs). The most conservative HBGVs were used to calculate risk characterisation ratios (RCRs) for each compound in a flexitarian, vegetarian and vegan scenario. If no HBGVs were available, NOAELs or BMDLs were divided by factor 500. RCRs below 1 indicate that the estimated exposure is unlikely to pose a significant health risk.

**Compound**	**EDI (ng/kg bw/day)**						**HBGV (ng/kg bw/day)**	**most sensitive endpoint**	**RCR flexitarian**	**RCR vegetarian**	**RCR vegan**
	Flexitarian scenario (10%)	Vegetarian scenario (50%)	Vegan scenario (100%)								
	PB meat alternative	PB meat alternative	PB meat alternative	PB fish alternative	PB cheese alternative	Sum vegan scenario					
TEP	0.01	0.1	0.1	0.03	0.1	0.2	1000000 [a]	repeated dose toxicity	1.0E-09	6.0E-08	2.1E-07
TCEP	0.004	0.02	0.04	0.01	0.02	0.1	7000 [b]	increased relative kidney weight	5.8E-07	2.9E-06	1.0E-05
TCIPP	0.1	0.7	1.3	2.1	0.6	4.1	10000 [b]	hepatocyte hypertrophy	1.3E-05	6.5E-05	4.1E-04
TiBP	0.04	0.2	0.4	1.1	1.1	2.7	2130000 [a]	repeated dose toxicity	2.0E-08	9.9E-08	1.3E-06
TDCIPP	0.2	1.2	2.4	0.6	1.1	4.2	17000 [a]	repeated dose toxicity	1.4E-05	7.1E-05	2.5E-04
TnBP	0.1	0.5	1.0	1.5	3.4	5.9	220000 [a]	repeated dose toxicity	4.6E-07	2.3E-06	2.7E-05
V6	0.0004	0.002	0.004	0.01	0.002	0.01	n.a.	n.a.	n.a.	n.a.	n.a.
TPhP	0.1	0.5	1.0	0.1	0.2	1.3	525000 [a]	repeated dose toxicity	2.0E-07	9.8E-07	2.4E-06
TBOEP	0.0	0.1	0.2	0.1	0.1	0.4	18000 [b*]	hepatic toxicity	1.1E-06	5.6E-06	1.9E-05
TDBPP	0.002	0.01	0.02	0.004	0.01	0.03	17580 [b*]	kidney tumors	9.2E-08	4.6E-07	1.6E-06
EHDPHP	1.8	8.8	18	2.6	0.8	21	36000 [a]	repeated dose toxicity	4.9E-05	2.5E-04	5.8E-04
TpTP	0.1	0.3	0.7	0.0	0.1	0.8	20000 [a]	repeated dose toxicity	3.4E-06	1.7E-05	4.0E-05
iDPP	0.1	0.3	0.7	0.2	0.1	1.0	3000000000 [b*]	developmental toxicity	2.2E-11	1.1E-10	3.2E-10
RDP	0.01	0.03	0.1	0.01	0.03	0.1	n.a.	n.a.	n.a.	n.a.	n.a.
TEHP	0.2	0.8	1.6	3.1	6.7	11	100000 [b]	developmental toxicity	1.6E-06	8.0E-06	1.1E-04
TBuPhP	0.01	0.03	0.1	0.003	0.005	0.1	n.a.	n.a.	n.a.	n.a.	n.a.
BDP	0.01	0.1	0.1	0.02	0.05	0.2	1670000 [a]	repeated dose toxicity	6.0E-09	3.0E-08	1.0E-07
**∑PFRs**	**2.7**	**14**	**27**	**12**	**14**	**53**					

[a] European Chemicals Agency ECHA [9], [b] U.S. Environmental Protection Agency US EPA [10], * HBGV not available, NOAEL or BMDL (obtained from US EPA) was divided by factor 500. N.a.: not available.

Table S 15. Exposure and risk assessment for individual plasticizers. Three separate scenarios were calculated: flexitarian (10% substitution of meat consumption with PB meat-alternatives), vegetarian (50% substitution of meat consumption with PB meat-alternatives) and vegan (100% substitution of meat, fish and cheese consumption with NPBFs). The most conservative HBGVs were used to calculate risk characterisation ratios (RCRs) for each compound in a flexitarian, vegetarian and vegan scenario. If no HBGVs were available, NOAELs or BMDLs were divided by factor 500. RCRs below 1 indicate that the estimated exposure is unlikely to pose a significant health risk.

**Compound**	**EDI (ng/kg bw/day)**						**HBGV (ng/kg bw/day)**	**most sensitive endpoint**	**RCR flexitarian**	**RCR vegetarian**	**RCR vegan**
	Flexitarian scenario (10%)	Vegetarian scenario (50%)	Vegan scenario (100%)								
	PB meat alternative	PB meat alternative	PB meat alternative	PB fish alternative	PB cheese alternative	Sum vegan scenario					
DMP	0.3	1.5	2.9	0.6	2.0	5.5	9400000 [a]	repeated dose toxicity	3.1E-08	1.6E-07	5.9E-07
DEP	2.3	12	23	3.6	21	48	750000 [a]	repeated dose toxicity	3.1E-06	1.5E-05	6.4E-05
DnBP	3.3	17	33	18	23	74	7000 [a]	developmental toxicity/teratogenicity	4.7E-04	2.4E-03	1.1E-02
DPP	0.0	0.1	0.2	0.1	1.2	1.4	n.a.	n.a.	n.a.	n.a.	n.a.
BBzP	0.2	0.9	1.9	0.1	2.9	4.9	200000 [b]		9.4E-07	4.7E-06	2.4E-05
DiBP	0.7	3.4	6.8	1.8	19	28	210000 [a]	developmental toxicity/teratogenicity	3.3E-06	1.6E-05	1.3E-04
DEHP	14	69	139	36	584	760	36000 [a]	carcinogenicity	3.9E-04	1.9E-03	2.1E-02
DINP	1.0	5.0	10	11	3.6	25	750000 [a]	repeated dose toxicity	1.3E-06	6.7E-06	3.3E-05
DIDP	0.8	4.0	8.1	1.1	5.9	15	750000 [a]	repeated dose toxicity	1.1E-06	5.4E-06	2.0E-05
ATEC	0.4	1.9	3.8	1.1	0.9	5.9		*no hazard identified*			
DIBA	0.2	0.9	1.9	0.5	0.7	3.1	n.a.	n.a.	n.a.	n.a.	n.a.
CDPHP	0.1	0.7	1.3	0.2	0.4	1.9	250000 [a]		5.3E-07	2.7E-06	7.6E-06
ATBC	1.5	7.6	15	2.4	56	73	1000000 [a]	repeated dose toxicity	1.5E-06	7.6E-06	7.3E-05
DBS	0.2	1.0	2.0	0.5	0.7	3.2		*no hazard identified*			
DEHA	0.3	1.5	3.0	0.8	13	17	1700000 [a]	repeated dose toxicity	1.8E-07	8.9E-07	1.0E-05
BTHC	0.004	0.02	0.04	0.002	0.05	0.1	1000000 [b*]	developmental toxicity	4.0E-09	2.0E-08	8.8E-08
THTM	0.001	0.00	0.01	0.002	0.1	0.2	n.a.	n.a.	n.a.	n.a.	n.a.
TOTM	0.02	0.1	0.2	0.1	0.1	0.4	1130000 [a]	repeated dose toxicity	2.1E-08	1.1E-07	3.7E-07
DEHT	4.8	24	48	13	432	493	3950000 [a]	repeated dose toxicity	1.2E-06	6.1E-06	1.2E-04
DINCH	0.8	3.8	7.7	2.0	3.0	13	2000000 [a]	carcinogenicity	3.8E-07	1.9E-06	6.3E-06
**∑LPs**	**23**	**113**	**225**	**73**	**663**	**961**					
**∑APs**	**8**	**42**	**83**	**20**	**507**	**610**					
**∑plasticizers**	**31**	**154**	**308**	**93**	**1169**	**1571**					

[a] European Chemicals Agency ECHA [9], [b] U.S. Environmental Protection Agency US EPA [10], * HBGV not available, NOAEL or BMDL (obtained from US EPA) was divided by factor 500. N.a.: not available.

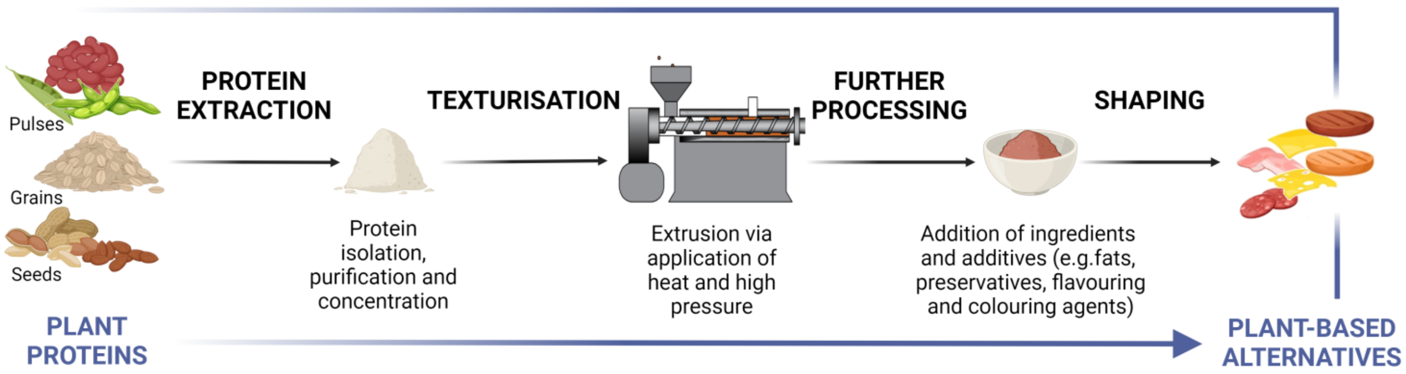

Figure S 1. Overview of industrial process steps for the production of novel plant-based foods. Created in BioRender. Poma, G. (2024) BioRender.com/t37h898

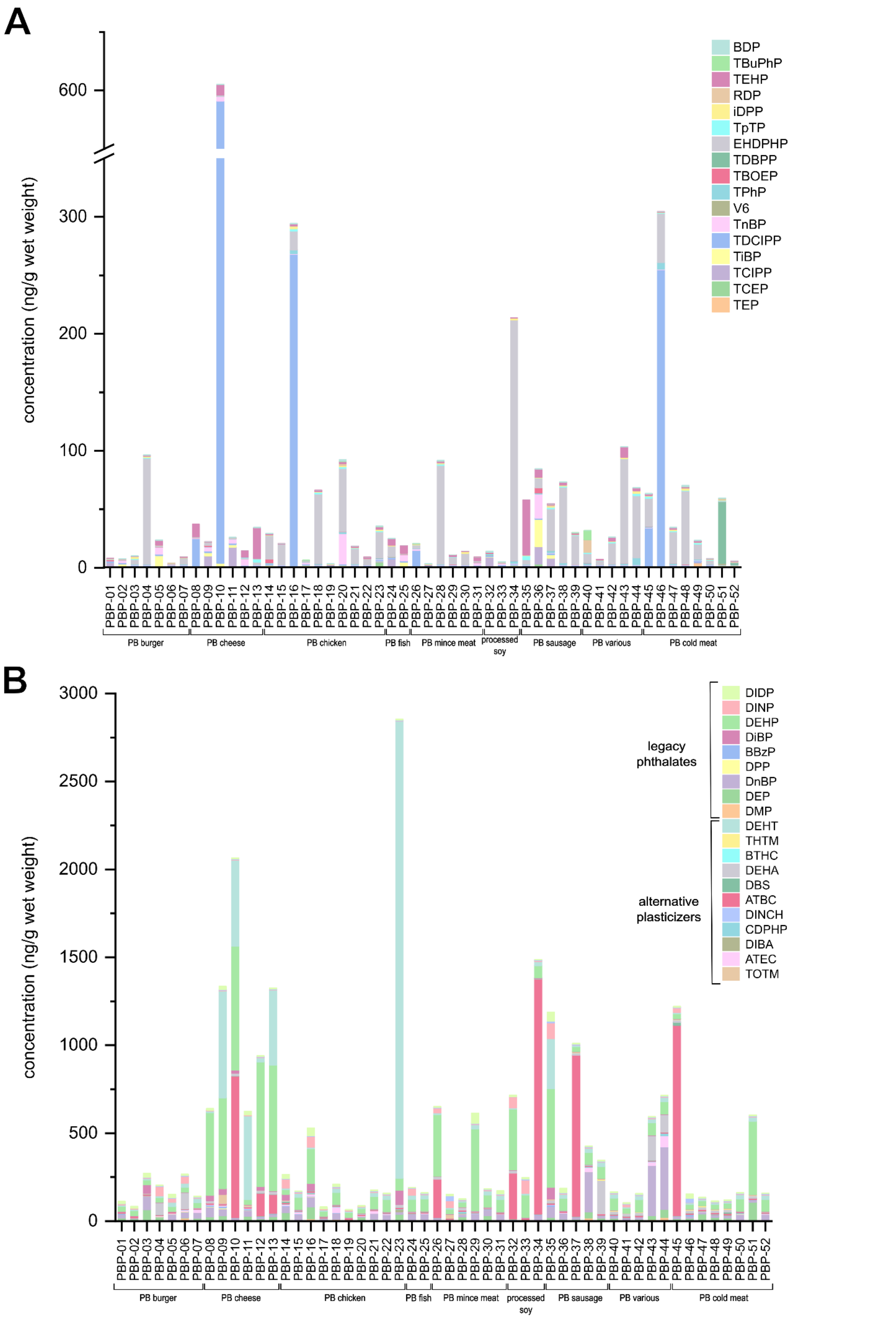

Figure S 2. Individual (A) PFR and (B) plasticizer contamination in ng/g wet weight per NPBF sample and contribution of compounds to the overall contamination.

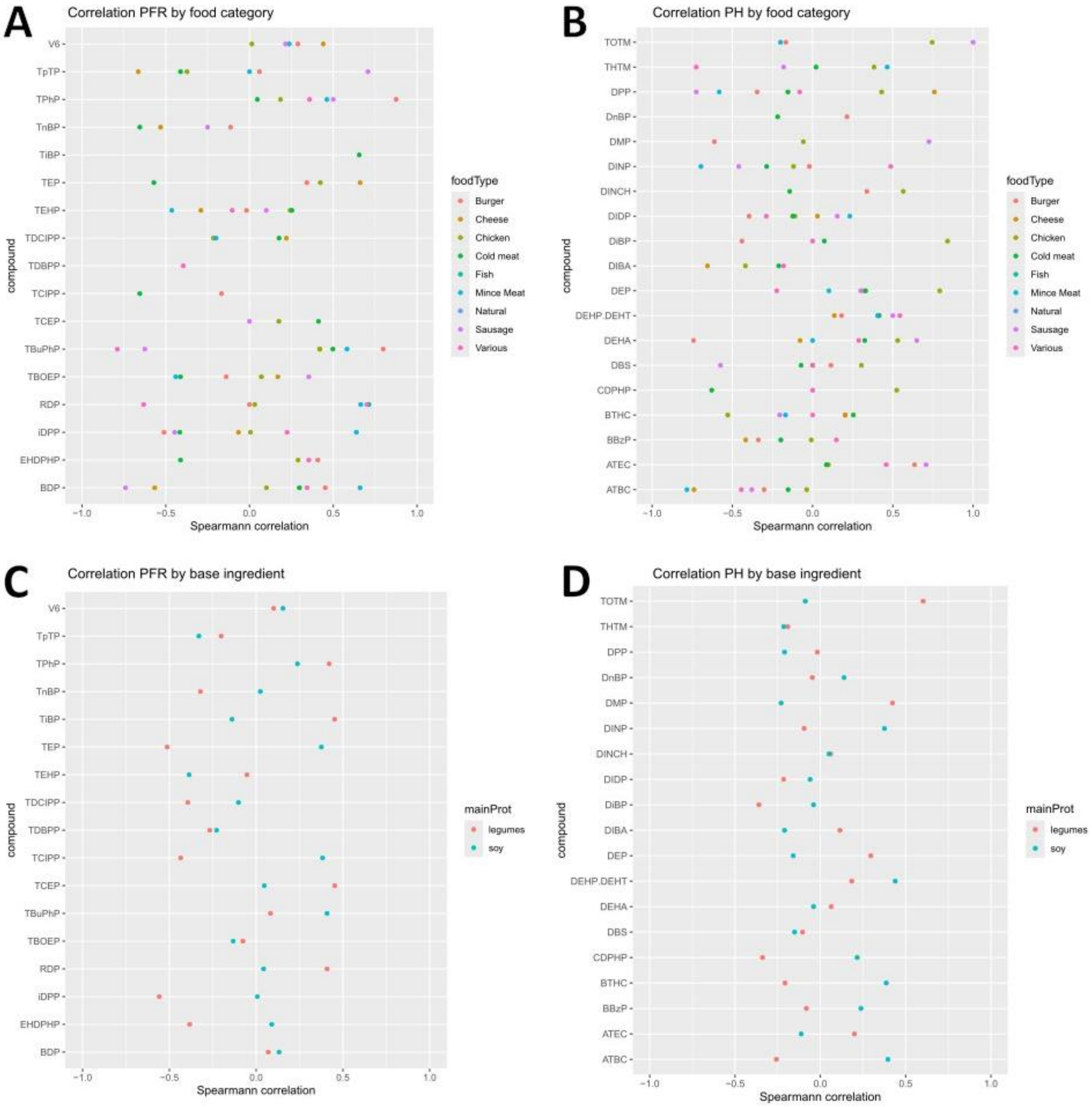

Figure S 3. Correlations between (A, B) FCM and NPBF contamination for individual PFRs and plasticizers according to food category and (C, D) base ingredient.

References

(1) Nájera Espinosa, S.; Hadida, G.; Jelmar Sietsma, A.; Alae-Carew, C.; Turner, G.; Green, R.; Pastorino, S.; Picetti, R.; Scheelbeek, P. Mapping the evidence of novel plant-based foods: a systematic review of nutritional, health, and environmental impacts in high-income countries. *Nutrition reviews* **2024.** DOI: 10.1093/nutrit/nuae031. Published Online: Apr. 25, 2024.

(2) Pointke, M.; Pawelzik, E. Plant-Based Alternative Products: Are They Healthy Alternatives? Micro- and Macronutrients and Nutritional Scoring. *Nutrients* **2022**, *14* (3). DOI: 10.3390/nu14030601. Published Online: Jan. 29, 2022.

(3) Las Heras-Delgado, S. de; Shyam, S.; Cunillera, È.; Dragusan, N.; Salas-Salvadó, J.; Babio, N. Are plant-based alternatives healthier? A two-dimensional evaluation from nutritional and processing standpoints. *Food research international (Ottawa, Ont.)* **2023**, *169*, 112857. DOI: 10.1016/j.foodres.2023.112857. Published Online: Apr. 19, 2023.

(4) Monteiro, C. A.; Cannon, G.; Levy, R. B.; Moubarac, J.-C.; Louzada, M. L.; Rauber, F.; Khandpur, N.; Cediel, G.; Neri, D.; Martinez-Steele, E.; Baraldi, L. G.; Jaime, P. C. Ultra-processed foods: what they are and how to identify them. *Public health nutrition* **2019**, *22* (5), 936–941. DOI: 10.1017/S1368980018003762. Published Online: Feb. 12, 2019.

(5) Zheng, L.; Regenstein, J. M.; Teng, F.; Li, Y. Tofu products: A review of their raw materials, processing conditions, and packaging. *Comp Rev Food Sci Food Safe* **2020**, *19* (6), 3683–3714. DOI: 10.1111/1541-4337.12640. Published Online: Oct. 3, 2020.

(6) Poma, G.; Fujii, Y.; Lievens, S.; Bombeke, J.; Gao, B.; Jeong, Y.; McGrath, T. J.; Covaci, A. Occurrence, patterns, and sources of hazardous organic chemicals in edible insects and insect-based food from the Japanese market. *Food and chemical toxicology : an international journal published for the British Industrial Biological Research Association* **2021**, *154*, 112311. DOI: 10.1016/j.fct.2021.112311. Published Online: Jun. 1, 2021.

(7) Poma, G.; Yin, S.; Tang, B.; Fujii, Y.; Cuykx, M.; Covaci, A. Occurrence of Selected Organic Contaminants in Edible Insects and Assessment of Their Chemical Safety. *Environmental health perspectives* **2019**, *127* (12), 127009. DOI: 10.1289/EHP5782. Published Online: Dec. 31, 2019.

(8) Christia, C.; Tang, B.; Yin, S.-S.; Luo, X.-J.; Mai, B.-X.; Poma, G.; Covaci, A. Simultaneous determination of legacy and emerging organophosphorus flame retardants and plasticizers in indoor dust using liquid and gas chromatography-tandem mass spectrometry: method development, validation, and application. *Analytical and bioanalytical chemistry* **2019**, *411* (26), 7015–7025. DOI: 10.1007/s00216-019-02078-5. Published Online: Sep. 12, 2019.

(9) ECHA. *ECHA chemicals database*. https://chem.echa.europa.eu/ (accessed 2024-07-25).

(10) USEPA. *CompTox Chemicals Dashboard*. https://comptox.epa.gov/dashboard/ (accessed 2024-07-25).
